# Machine learning approaches to predict early cardiac immune-related adverse events in patients receiving immune checkpoint inhibitors

**DOI:** 10.1007/s00520-026-10984-5

**Published:** 2026-07-29

**Authors:** Michael Sayer, Peter D. Chang, Hirofumi Hamano, Reina Yamamoto, Misako Nagasaka, Ali A. Naqvi, Pranav M. Patel, Yoshito Zamami, Aya F. Ozaki

**Affiliations:** 1 School of Pharmacy & Pharmaceutical Sciences, University of California, Irvine, CA USA; 2 School of Medicine, Pathology & Laboratory Medicine, University of California, Irvine, CA USA; 3 Donald Bren School of Information and Computer Sciences, University of California, Irvine, CA USA; 4 Department of Pharmacy, Medical Development Field, Okayama University, Okayama, Japan; 5 Division of Hematology and Oncology, University of California, Irvine, CA USA; 6 Mary & Steve Wen Cardiovascular Division, Department of Medicine, University of California, Irvine, CA USA; 7 Department of Medicinal Pharmacology, Graduate School of Medicine, Dentistry, and Pharmaceutical Sciences, Okayama University, Okayama, Japan

**Keywords:** Immune checkpoint inhibitors, Immune-related adverse events, Myocarditis, Pericarditis, Machine learning, Risk assessment tools

## Abstract

**Purpose:**

Immune checkpoint inhibitor (ICI)–induced cardiac immune-related adverse events (cardiac irAEs) are rare yet serious complications. Clinical assessment tools to identify at-risk patients would allow for more effective prevention strategies, thus improving clinical outcomes. We constructed various machine learning (ML) models to predict these events among patients receiving ICI therapy.

**Methods:**

A cohort of patients receiving ICI therapy from 2010 to 2023 was identified from the TriNetX database. Cardiac irAEs were defined as the occurrence of relevant diagnosis codes within 90 days of ICI initiation, with corresponding hospital visits. We created ML models to predict these events, including elastic net logistic regression and multiple tree-based approaches (gradient boosted trees and random forest). We evaluated model performance with different performance measures and utilized assigned risk scores to stratify risk of cardiac irAEs into low, medium, and high-risk tiers.

**Results:**

We identified 61,117 patients receiving ICI therapy, with nearly 2% of patients experiencing cardiac irAEs. Model performance on testing data was comparable with all approaches (AUC = 0.71–0.72, balanced accuracy = 65–66%). Each model emphasized distinct features to make classifications, as observed with feature importance and SHAP values. Comparing cardiac irAE rates among assigned risk strata, patients identified as high risk were significantly more likely to experience cardiac irAEs compared to lower tiers.

**Conclusion:**

Our preliminary exploration of ML methods demonstrated the potential for risk assessment tools to predict rare cardiac irAEs in patients receiving ICI therapy. Follow-up studies can implement time series approaches to harness longitudinal data that incorporates real-time labs, new diagnoses, and new therapy, to refine predictions further.

**Supplementary Information:**

The online version contains supplementary material available at 10.1007/s00520-026-10984-5.

## Introduction

With rapidly growing use of immune checkpoint inhibitor (ICI) therapy in cancer treatment, continued research improving the ability to identify patients at risk for immune-related adverse events (irAEs) is vital. ICI agents function by blocking signaling checkpoints that restrict immune activity against cancer cells but may affect healthy tissue as well, leading to unwanted inflammation in various organ systems and manifesting a variety of complications [1]. Each systemic class of irAEs has distinct risk factors, necessitating that studies emphasize specific classes of irAEs when deriving clinically meaningful predictive tools [2]. One class of irAEs that is particularly challenging to evaluate is cardiac irAEs [3].

ICI-induced cardiac irAEs are a rare yet serious complication of ICI therapy. Previous literature has characterized cardiac irAEs as the onset of myocarditis in ICI patients, occurring in less than 1% of the population [4, 5]. ICI-induced myocarditis leads to worsened cancer treatment outcomes, with studies highlighting ~ 25% increase in mortality risk for 1-year survival endpoints [3, 6, 7]. Considering broader cardiac inflammation diagnoses (i.e., pericarditis, pericardial diseases) significantly expands the population impacted by these serious events, as newer research has shown that ICI-induced pericarditis has comparable negative treatment outcomes [3, 8]. Cardiac irAE timing relative to ICI initiation onset has clinical implications, with researchers suggesting “early onset” events being more severe and having a distinct etiology [9, 10]. Definitions of this “early onset” window tend to range from 90 to as much as 180 days, also influenced by time removed from last ICI treatment exposure [11–13]. Clinical management of cardiac irAEs is a unique challenge, as the many guideline-based interventions can complicate cancer treatment approaches [14, 15]. Identification of risk factors for cardiac irAEs and the subsequent development of risk assessment tools would allow clinicians and patients to gauge their risk for these serious events.

Machine learning (ML) algorithms have the potential to mitigate challenges researching cardiac irAEs. Many ML modeling strategies can better manage rarer outcomes, as they can incorporate resampling techniques and are less susceptible to instability caused by violations of traditional statistical assumptions [16, 17]. ML model training approaches can prioritize and consider many different potential combinations of predictive characteristics, in addition to harnessing larger trends that are not possible with conventional modeling strategies [18]. Researchers have embraced these approaches to identify risk factors for and make effective predictions of cancer treatment–based toxicities in other settings, with developed models even being the basis of risk assessment tools now used in practice [19–21]. Building ML models in which electronic health record (EHR) data predicts cardiac irAEs can lead to many new insights and eventual improvement of risk assessment evaluations for patients starting ICI therapy. It may also assist in identifying patient populations best served with closer surveillance and initiation of cardioprotective therapies.

 With this study, we explored ML approaches in assessing cardiac irAE risk in ICI patients, emphasizing routinely available information in electronic health records at the time of ICI initiation. ML techniques are particularly well suited to this context given their ability to process high-dimensional clinical data, identify relevant predictors, and address the class imbalance associated with rare outcomes. Leveraging the large and diverse real-world population within the TriNetX database enabled broad representation of clinical and demographic characteristics, enhancing the generalizability of model findings. Our study aimed to develop and evaluate the feasibility of scalable, EHR-based ML models for identifying patients at elevated risk of cardiac irAEs, with the goal of supporting future risk stratification efforts and advancing personalized care in cardio-oncology.

## Methods

### Study design and patient population

We performed a multicenter, retrospective cohort study, evaluating patients within the TriNetX platform who received ICI therapy [22]. Files of patient data across 115 institutions were obtained on October 28, 2024, which included all patients receiving ICI therapies from January 1, 2010, to December 31, 2023.

### Index immune checkpoint inhibitor(s), index date, and time on therapy

ICI index date was the first date of ICI administration in each patient’s medical record, while index ICI agent was based on ICI agents given on a patient’s index date. Index agents were grouped by mechanistic class, including anti-PD-1 monotherapy (nivolumab, pembrolizumab, cemiplimab, dostarlimab), anti-PD-L1 monotherapy (atezolizumab, avelumab, durvalumab), anti-CTLA-4 monotherapy (ipilimumab), and combination ICI therapy (pembrolizumab + ipilimumab, nivolumab + ipilimumab). Time on ICI therapy was defined as the number of days between the ICI index date and the last recorded instance of ICI therapy in the patient’s medical record.

### Cardiac inflammation

Cardiac inflammation was defined by International Classification of Diseases, Ninth (ICD-9) and Tenth (ICD-10) Revision codes (Supplemental Table 1) [23–25]. This included diagnostic codes representative of myocarditis (I41.1, I41.8, I41.9, I51.4, I41), pericarditis (I30.0, I30.8, I30.9, I32), and pericardial diseases (I31.2, I31.3, I31.4, I31.8, I31.9) consistent with precedent literature (Supplemental Table 1). Prior cardiac inflammation was defined as the occurrence of these diagnoses up to 90 days prior to and including the ICI index date. Adverse events within the study were defined as the first occurrence of selected diagnosis codes within 90 days of index date, excluding day of index (Supplemental Figure 1) [10, 11]. Additional criteria further defined ICI-induced cardiac irAEs in our study. First, the diagnosis had to occur no more than 30 days after the last appearance of ICI therapy in a patient’s medical record to ensure an association with ICI administration. Second, the patient had to have an accompanying hospital encounter within 1 week of its occurrence. This hospitalization requirement was intended to capture clinically significant events and reduce reliance on diagnostic coding alone when defining cardiac irAEs. Identified events not meeting these criteria, including not occurring within the defined window relative to last documented ICI and/or lacking an accompanying hospitalization, were considered “ambiguous cardiac inflammation diagnoses” and not adverse events in the study.

### Inclusion/exclusion criteria

Inclusion criteria for the study included receipt of ICI therapy from January 2010 to December 2023. Additionally, patients were included if they experienced a cardiac irAE or had at least 60 days of time on ICI therapy. Patients were excluded if they lacked a concurrent neoplasm diagnosis or were missing biological sex and/or birth date information. Patients with less than 60 days on ICI therapy who did not experience a cardiac irAE were excluded, as an event-free classification in this group may reflect insufficient exposure and observation time rather than a true absence of cardiac irAE. Finally, patients experiencing prior cardiac inflammation or ambiguous cardiac inflammation diagnoses were also excluded from the study.

### Patient data

Patient age at index was represented with four categorical groupings including age 18 to 44, age 45 to 64, age 65 to 74, and ages 75 and older. Patient race and ethnicity was characterized as one of the following: non-Hispanic White, non-Hispanic Black, non-Hispanic Asian, Hispanic, or unknown. Primary cancer type was characterized based on the first appearance of malignancy diagnosis codes up to 1 year prior to ICI initiation, including 22 distinct primary cancer types. Primary cancer types were clusters of ICD-9 and10 codes representative of different primary malignancies. Patients with diagnosis codes associated with multiple different primary cancer types at the time of their first cancer diagnosis or having only nonspecific malignancy diagnoses were labeled as “other” cancer types.

Patient comorbidities were based on those included within the Elixhauser Comorbidity Index, which included 31 different comorbid conditions. Patients were considered to have a given comorbidity if they had a relevant diagnosis code up to 1 year preceding ICI initiation (Supplemental Figure 1) [26–28]. We used the National Cancer Institute (NCI) comorbidity index as cumulative index of comorbidity burden [29]. Comorbidity burden was subsequently categorized into tertiles as “low,” “medium,” or “high,” with “high”-based population ranking. To characterize medication exposures, we selected 250 + different Anatomical Therapeutic Chemical (ATC) class codes referencing the World Health Organizations (WHO) center of drug statistics methodology database [30]. Our primary dataset provided by TriNetX utilized a combination of ATC codes to characterize medication classes and RxNorm codes to designate individual agents. Specific ATC classes were grouped in consultation with clinicians to reflect pharmacologic mechanisms, indications, and expected prevalence in the patient population. Patients were considered to have used a given ATC class if they had documented use of any agent within that class up to 90 days prior to index date (Supplemental Figure 1).

Greater detail describing the source and generation of each element of patient data requisitioned from TriNetX is included in Supplemental Methods section 1. Management of missing data was feature dependent. Individuals with missing age, biological sex, or cancer diagnoses were excluded from the study. For all other features, missing values were interpreted as patients not experiencing a given medication exposure or previous diagnosis.

### Machine learning approaches implemented

Features considered for ML models included patient demographic information (age, sex, race/ethnicity), ICI index agent (PD-1 monotherapy, PD-L1 monotherapy, CTLA-4 monotherapy, and combination therapy), NCI comorbidity index (low, medium, or high), primary cancer diagnosis (22 different categories), comorbid conditions (31 different conditions), and past medication exposures (253 ATC classes). To ensure stability of model estimates, we restricted inclusion to primary cancer diagnoses, comorbid conditions, and medication exposures that occurred in at least 20 patients in the cohort who experienced a cardiac irAE from the cumulative dataset. 

Patient data was then separated into training and testing data via a random 70:30 split stratified by occurrence of cardiac irAEs (Supplemental Figure 2). Three different ML approaches were employed to predict the occurrence of cardiac irAEs, including elastic net logistic regression, gradient boosted trees, and random forests (Supplemental Table 2) [31, 32]. For each approach, two training steps were implemented, with initial models evaluating sampling techniques and implement feature pruning. A final model was then created with the selected sampling strategy and features. With both steps, a broad hyperparameter space specific to each model type was used, with *G*-means as the optimization parameter. *G*-means was initially selected to achieve the best balance of sensitivity and specificity for binary classification of a rare outcome. *G*-means maximizes overall model discriminatory power independent of disease prevalence while other measures (i.e., *F*1-score, PR-AUC) can have limitations with rarer outcomes. Model performance measures across the hyperparameter space were assessed by means of threefold cross-validation with 10 repeats. The combination of hyperparameters with the highest average *G*-means score across all folds was selected and utilized to train the model with all training data (Supplemental Table 2, Supplemental Figure 2).

### Training machine learning models

Initially, eight different sampling techniques were implemented to evaluate optimal sampling approaches for cardiac irAE classification (Supplemental Table 3, Supplemental Figure 2). The sampling approach achieving the highest average *G*-means score across all folds was selected, as it accounts for both sensitivity and specificity when evaluating performance in imbalanced datasets. The models using the selected sampling approach were subsequently used for feature pruning, where features having minimal importance (a value of 0 in feature importance measure) were selected for removal. The final models were trained with the selected sampling approach and features.

### Identification of critical features for cardiac irAE prediction

With the final, trained models, the contributions of key features were characterized (Supplemental Figure 2). First, scaled feature importance values were collected. Next, SHAP values were determined utilizing a game theory approach, where Monte Carlo simulations generated SHAP values using training data and created models [33, 34]. For comparison, the top 20 features for each model, as characterized by highest feature importance values, were reviewed. Specific to each model, features among the top 10 for feature importance and mean SHAP values were also highlighted. Mean absolute SHAP value plots further characterized the contribution of key features [35].

### Model predictions

Each model was utilized to predict cardiac irAEs in the training and testing datasets, with multiple performance metrics characterizing performance (Supplemental Figure 2). For final models, we further implemented a nonparametric bootstrap with replacement approach, resampling data 1000 times. Predictions were made for each bootstrap replicate, and performance measures were summarized using 95% confidence intervals. As a means of comparison, performance measures for “Null Model” predictions were also captured, predicting all cases to be noncardiac irAEs, representing the baseline majority class prediction.

To better understand the relationship between predicted probabilities and observed adverse event rates, calibration curves were created from each model on testing data. Predicted probabilities were grouped into five quantiles, and the observed adverse event rate within each quantile was calculated. Fitted trend lines were then generated by plotting the mean predicted probability against the observed event rate. Assigned probabilities were further used to indicate risk tiers for clinical utility. Low-risk patients were those with assigned probabilities less than 0.5. Medium and high-risk categorization was specific to each model, using a quantile-based approach. The top 10% of the highest assigned probabilities (above 0.5) were assigned to the high-risk category, while the rest were medium risk. We compared incidence of cardiac irAEs between risk tiers with chi-square test and Fisher’s exact test (high vs medium/low).

## Results

### Study population

Within TriNetX, we identified 113,572 potential patients who received ICI therapy from January 1 st, 2010, to December 31 st, 2023. Of these patients, 61,117 were eligible to be included in the study, with patients excluded for not meeting eligibility criteria including: no neoplasm diagnosis (*n* = 4120), no biological sex (*n* = 5487) or date of birth information (*n* = 2127), younger than 18 years of age (*n* = 296), having prior cardiac inflammation diagnoses (*n* = 4690), having ambiguous cardiac inflammation occurrence (*n* = 987), or insufficient time on ICI therapy (*n* = 34,748) (Table 1, Supplemental Figure 3). Among included patients in the study, 1172 (1.9%) experienced a cardiac irAE (Table 1). The average time to cardiac irAE was 33 days (SD = 24 days), with 54.3% (*n* = 636) of these events occurring within 30 days of ICI initiation (Supplemental Figure 4).

**Table 1:** This table presents study population demographic characteristics. Categorical features describe proportions of the patient population (percentage (count)). Numeric features describe averages across the patient population (mean (standard deviation)). For primary cancer type, ICD-10 codes associated with each primary cancer type are included in parentheses

Category	Feature	Statistic
**Total patients**	Patients meeting inclusion/exclusion criteria	**61,117 patients**
**Adverse events**	*Total cardiac irAEs*	1.9 (1172)
	*Time to adverse event (days) [mean (SD)]*	33.3 (24.0)
**Age**	*Age (years) [mean (SD)]*	64.5 (12.5)
	*Age 18 to 44*	7.3 (7047)
	*Age 45 to 64*	37.4 (22,859)
	*Age 65 to 74*	33.2 (20,311)
	*Age 75 and up*	21.8 (13,321)
**Sex**	*Male*	56.7 (34,637)
**Race/ethnicity**	*White*	72.6 (44,345)
	*Black*	9.0 (5491)
	*Hispanic*	4.5 (2746)
	*Asian*	4.2 (2590)
**ICI index agent**	*PD-1 agent*	73.3 (44,797)
	*PD-L1 agent*	17.1 (10,476)
	*CTLA-4 agent*	2.5 (1531)
	*Combination therapy*	7.1 (4313)
**NCI comorbidity score**	*NCI score [mean (SD)]*	0.57 (0.63)
	*Low NCI score*	35.2 (21,507)
	*Medium NCI score*	34.2 (20,881)
	*High NCI score*	30.6 (18,729)
**Primary cancer type**	Lung (C34)	26.2 (15,986)
	Skin (C43–C44)	15.7 (9595)
	Kidney (C64)	7.3 (4478)
	Other digestive (C15–C17, C21, C23–C26)	5.4 (3287)
	Breast (C50)	5.3 (3256)
	Liver (C22)	4.2 (2589)
	Oral (C0–C14)	4.1 (2509)
	Bladder (C67)	4.1 (2503)
	Blood (C81–C96)	2.7 (1619)
	Uterine (C54–C55)	2.4 (1476)
	Colorectal (C18–C20)	2.3 (1380)
	Prostate (C61)	1.9 (1135)
	Mesothelial soft tissue (C45–C49)	1.8 (1068)
	Other respiratory (C30–C33, C35–C39)	1.6 (967)
	Central nervous system (C69–C72)	1.3 (811)
	Cervical (C53)	1.1 (690)
	Other genitourinary (C65–C66, C68)	0.9 (539)
	Male other (C60, C62–C63)	0.8 (515)
	Ovarian (C56)	0.8 (475)
	Endocrine (C73–C75)	0.6 (335)
	Female other (C51–C52, C57–C58)	0.5 (319)
	Bone (C40–C41)	0.4 (228)
	Other/nonspecific	4.8 (2920)
**Machine learning datasets**	Training data	70.0 (42,782)
	Testing data	30.0 (18,335)

Average patient age was 64.5 years old (SD = 12.5 years) with most patients either being 65 to 74 years old (33.2%) or 75 and older (21.8%) (Table 1). Additionally, most patients identified as non-Hispanic White (72.6%) and received anti-PD-1 monotherapy (73.2%) (Table 1, Supplemental Table 14). Lung (26.2%) and skin (15.7%) cancers were the most commonly observed primary cancer types, with other types occurring in less than 10% of the patient population and 4.8% of the population having nonspecific cancer diagnoses (Table 1, Supplemental Table 14). Most patients had metastatic cancer (67.8%), in addition to comorbid cardiac arrhythmia (23.7%), uncomplicated hypertension (37.1%), fluid/electrolyte disorder (22.0%), and chronic pulmonary disease (24.5%) (Supplemental Table 5). The top observed medication exposures, occurring in at least 50% of the patient population, included opioids (N02A) (63.0%), 5HT3 serotonin antagonists (A04AA) (66.4%), corticosteroids (D07) (66.0%), anilides (N02BE) (52.0%), and antihistamines (R06A) (51.2%) (Supplemental Table 6). Additional patient characteristic details, including frequency comparisons among irAE and non-irAE patients, can be found in Supplemental Tables 4–6.

### Machine learning data

Initial variable selection based on prevalence among cardiac adverse event patients (at least 20 patients) led to inclusion of 13 of 22 primary cancer types, all 31 potential comorbid conditions, and 128 of 253 potential medication exposures, in addition to all patient demographic characteristics (Supplemental Tables 4–6). Next, 61,117 eligible patients were randomly assigned to training (*n* = 42,782) and testing (*n* = 18,335) datasets by means of a 70:30 split stratified by adverse event (Table 1, Supplemental Tables 4–6).

### Elastic net logistic regression

With initial modeling, downsampling achieved the highest *G*-means value and 43 features were selected for final model training (Supplemental Table 7, Supplemental Table 8). Using the final model to make predictions, performance metrics with testing data slightly declined relative to those observed on the training dataset (*G*-means 0.693 (95% CI 0.677–0.709) training vs 0.653 (95% CI 0.624–0.679) testing, AUC 0.751 (95% CI 0.734–0.768) training vs 0.718 (95% CI 0.691–0.742) testing). Observed performance measures for final model predictions were comparable to those observed with the initial model (Table 2, Supplemental Table 9). With assigned risk tiers, high-risk patients were significantly more likely to experience a cardiac irAE (9.2% of patients) compared to those assigned medium risk (3.5%) or low risk (1.1%) tiers (Table 3).

**Table 2:** This table presents predictive performance of final models with training and testing data. From left to right, the first column lists the machine learning model used, followed by the selected hyperparameters for each model, an indication of training or testing dataset for subsequent reported results, and different performance metrics utilized. Reported results are 95% confidence intervals for each measure described

**Model**	**Elastic net**		**Gradient boosted trees**		**Random forest**		**Null model**	
Tuning	Alpha = 0.4 Lambda = 0.00464		Maximum depth = 5 Learning rate (ETA) = 0.1 Minimum child weight = 40		MTRY = 40 Minimum node size = 20			
Dataset	Training	Testing	Training	Testing	Training	Testing	Training	Testing
AUC	0.751 (0.734–0.768)	0.717 (0.691–0.742)	0.767 (0.751–0.782)	0.718 (0.692–0.745)	0.928 (0.918–0.938)	0.719 (0.691–0.746)	0.500	0.500
PRAUC	0.065 (0.056–0.077)	0.058 (0.047–0.074)	0.074 (0.064–0.087)	0.054 (0.044–0.07)	0.411 (0.377–0.447)	0.058 (0.047–0.075)	0	0
Sensitivity	0.672 (0.640–0.703)	0.599 (0.546–0.645)	0.731 (0.699–0.761)	0.659 (0.614–0.708)	0.948 (0.933–0.963)	0.692 (0.643–0.74)	0	0
Specificity	0.715 (0.700–0.719)	0.711 (0.705–0.717)	0.667 (0.663–0.672)	0.656 (0.649–0.664)	0.641 (0.636–0.645)	0.631 (0.624–0.638)	1	1
*G*-means	0.693 (0.677–0.709)	0.653 (0.624–0.679)	0.699 (0.683–0.713)	0.658 (0.634–0.682)	0.779 (0.772–0.786)	0.661 (0.636–0.684)	NA	NA
Accuracy	0.714 (0.710–0.718)	0.709 (0.703–0.715)	0.669 (0.664–0.673)	0.656 (0.649–0.664)	0.646 (0.642–0.651)	0.632 (0.625–0.639)	0.980	0.980
Balanced accuracy	0.693 (0.678–0.709)	0.655 (0.629–0.679)	0.699 (0.683–0.714)	0.658 (0.634–0.682)	0.794 (0.786–0.802)	0.662 (0.636–0.686)	0.500	0.500
*F*1	0.082 (0.075–0.088)	0.075 (0.066–0.085)	0.077 (0.071–0.083)	0.071 (0.063–0.079)	0.092 (0.086–0.098)	0.07 (0.062–0.078)	0.020	0.020
Positive predictive value	0.043 (0.040–0.047)	0.04 (0.035–0.045)	0.041 (0.037–0.044)	0.037 (0.033–0.042)	0.048 (0.045–0.052)	0.037 (0.033–0.041)	0.000	0.000
Negative predictive value	0.991 (0.990–0.992)	0.989 (0.987–0.99)	0.992 (0.991–0.993)	0.99 (0.988–0.991)	0.998 (0.998–0.999)	0.99 (0.988–0.992)	0.980	0.980

**Table 3:** This table presents cardiac immune-related adverse event risk among assigned risk tiers from each ML model. From left to right, the first column lists the machine learning model used, followed by the named risk tier being described in the adjacent column. Next, the total number of patients (total patients) within each prospective tier is shown, followed by the total patients within that tier experiencing a cardiac irAE (total AEs) and the percentage of patients within that tier that had the adverse event (percent AEs). The last two columns describe statistical tests comparing proportions of patients experiencing irAEs; first is “Fisher’s test” comparing proportions of low and medium tier groups to high in separate tests. Last, a cumulative chi-square test compares proportions across all three groups

	**Risk tier**	**Total patients (*n*)**	**Total AEs (*n*)**	**Percent AEs (%)**	**Fisher’s test**	**Chi-square test**
Elastic net	*Low*	12,924	146	1.13	1.01e−08	< 2.2e−16
	*Medium*	4870	168	3.45	< 2.2e−16	
	*High*	541	50	9.24		
Gradient boosted trees	*Low*	11,920	124	1.04	2.45e–05	< 2.2e–16
	*Medium*	5773	195	3.38	< 2.2e–16	
	*High*	642	45	7.01		
Random forest	*Low*	11,453	112	0.98	8.53e–12	< 2.2e–16
	*Medium*	6193	190	3.07	< 2.2e–16	
	*High*	689	62	9.00		

**Fig. 1:**
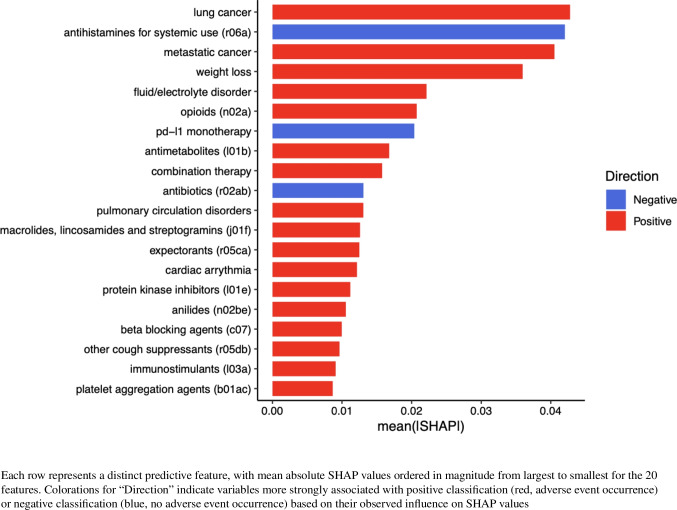
This bar chart represents mean absolute SHAP values for top 20 features of elastic net logistic regression model

Among the model’s top 20 features, 11 were medication exposures, 3 were specific comorbid conditions, 3 were related to cancer diagnoses, and the remaining were associated with different baseline characteristics (Supplemental Table 14, Supplemental Table 10). With respect to SHAP values, all features except for 3 were more strongly associated with positive prediction among the top 20 highlighted (Fig. 1). Features within the top 10 with respect to mean SHAP value and feature importance magnitude included lung cancer diagnosis, combination ICI therapy, and antibiotics (R02AB) (Supplemental Table 10).

### Gradient boosted trees

With initial modeling, downsampling achieved the highest *G*-means value and 62 features were selected for final model training (Supplemental Table 7, Supplemental Table 8). Using the final model to make predictions, performance metrics with testing data slightly declined relative to those observed on the training dataset (*G*-means 0.699 (95% CI 0.683–0.714) training vs 0.658 (95% CI 0.634–0.682) testing, AUC 0.767 (95% CI 0.751–0.782) training vs 0.718 (95% CI 0.692–0.742) testing). Observed performance measures for the final model were comparable to those observed with the initial model (Table 2, Supplemental Table 9). High-risk patients were significantly more likely to experience a cardiac irAE (7.0% of patients) compared to those assigned medium-risk (3.4%) or low-risk (1.0%) tiers (Table 3).

**Fig. 2:**
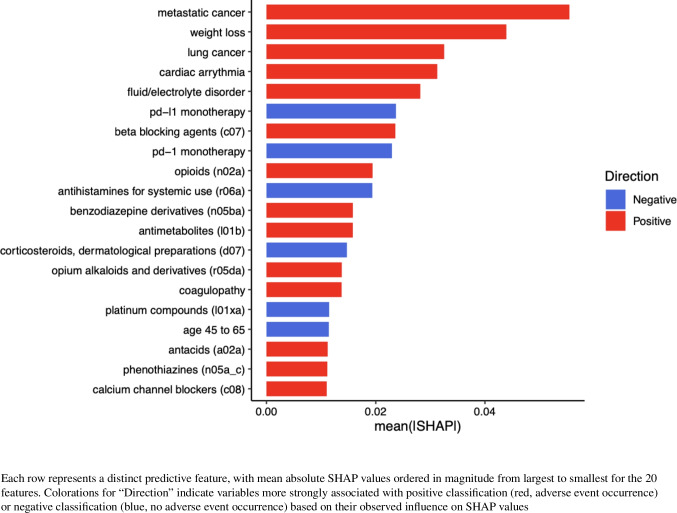
This bar chart represents mean absolute SHAP values for top 20 features of gradient boosted trees model

Among the model’s top 20 features, 9 were medication exposures, 6 were specific comorbid conditions, 2 were related to cancer diagnoses, and the remaining were associated with different baseline characteristics (Supplemental Table 14, Supplemental Table 11). With respect to SHAP values, all features except 6 were more strongly associated with positive prediction among the top 20 highlighted (Fig. 2). Features within the top 10 with respect to mean SHAP value and feature importance magnitude included metastatic cancer, weight loss, lung cancer, cardiac arrhythmia, fluid/electrolyte disorder, beta blocking agents (C07), and PD-1 monotherapy (Supplemental Table 11).

### Random forest

With initial modeling, downsampling achieved the highest *G*-means value with 186 selected for final model training (Supplemental Table 7, Supplemental Table 8). Using the final model to make predictions, performance metrics with testing data significantly declined relative to those observed on the training dataset (*G*-means 0.779 (95% CI 0.772–0.786) training vs 0.661 (95% CI 0.636–0.684) testing, AUC 0.928 (95% CI 0.918–0.938) training vs 0.719 (95% CI 0.691–0.746) testing). Observed performance measures for final modeling were comparable to those observed with the initial model (Table 2, Supplemental Table 9). High-risk patients were significantly more likely to experience a cardiac irAE (9.0% of patients) compared to those assigned medium-risk (3.1%) or low-risk (0.98%) tiers (Table 3).

**Fig. 3:**
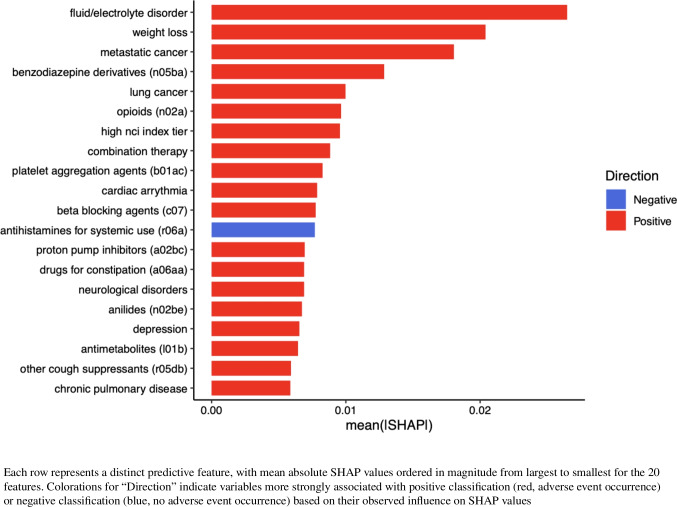
This bar chart represents mean absolute SHAP values for top 20 features of random forest model

Among the model’s top 20 features, 8 were medication exposures, 6 were specific comorbid conditions, 2 were related to cancer specific diagnoses, and the remaining were associated with different baseline characteristics (Supplemental Table 14, Supplemental Table 12). With respect to SHAP values, all features except 1 were more strongly associated with positive prediction among the top 20 highlighted (Fig. 3). Features within the top 10 with respect to SHAP value and feature importance magnitude included weight loss, fluid/electrolyte disorder, metastatic cancer, cardiac arrhythmia, high NCI index tier, opioids (N02A), combination therapy, and benzodiazepine derivatives (N05BA) (Supplemental Table 12).

### Model comparison

**Fig. 4:**
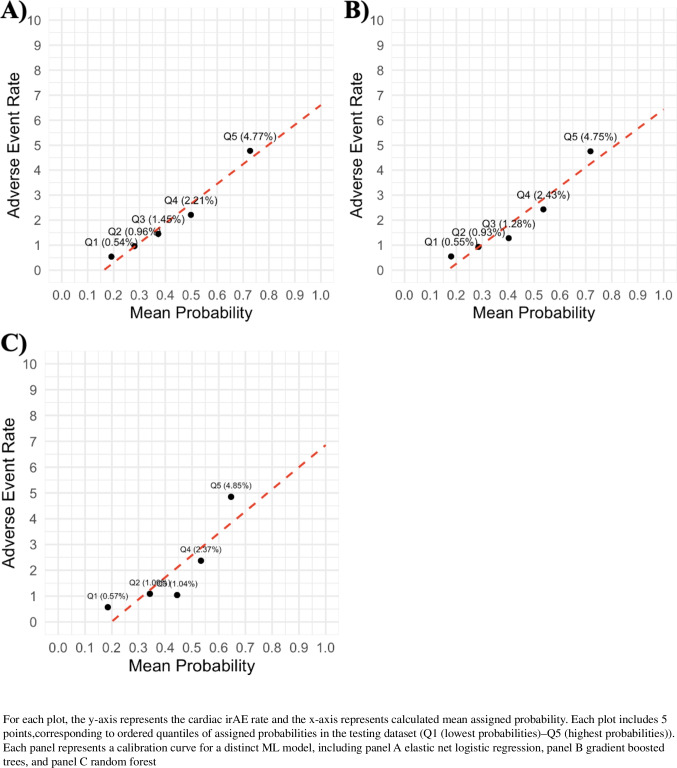
Linear plots representing calibration curves for ML models

Model performance with respect to testing dataset predictions was consistent across the three models, when comparing *G*-means, AUC, and balanced accuracy metrics (Table 2). When implementing them as a risk assessment tool, it appeared the elastic net logistic regression and random forest models had superior performance, especially when comparing cardiac irAE incidence between high risk and medium risk tiers (Table 3). Considering calibration curves, fitted trend lines for all models appear to be consistent with expected linear trends (Fig. 4, Supplemental Table 13). Furthermore, the fit for random forest had significantly more variance, showing an erratic nonlinear pattern for points representing early quantiles (Fig. 4, Supplemental Table 13). For all calibration curves, the quantile representing the highest assigned probabilities (top 20% of assigned probabilities) show significantly higher adverse event rates relative to lower quantiles, thereby deviating from expected linear increases (Fig. 4, Supplemental Table 13). Adverse event rates observed within quantiles and calibration curves suggest underprediction of risk relative to expected for all models (Fig. 4, Supplemental Table 13). This can be due to limitations with retrospective data collection, where patients experiencing adverse events have incomplete health histories within the database, leading to lower assigned risk scores. Also, patients experiencing cardiac irAEs may have other predisposing risk factors not captured by our dataset, leading to lower assigned risk scores than expected.

Among the three models, 4 features overlapped within their perspective top 20 features, including metastatic cancer, weight loss, lung cancer, and pulmonary circulation disorders (Supplemental Table 14). Furthermore, there were 7 additional overlapping features between gradient boosted tree and random forest models, including cardiac arrhythmia, fluid/electrolyte disorder, beta blocking agents (C07), benzodiazepine derivatives (N05BA), chronic pulmonary disease, and opioids (N02A) (Supplemental Table 14). Only 1 feature for elastic net overlapped with gradient boosted trees (antihistamines for systemic use (R06A)) and 2 overlapped with random forest models (combination ICI therapy, other cough suppressants (R05DB)).

## Discussion

With our study, we collected a cohort of over 60,000 patients receiving ICI therapy that met our inclusion/exclusion criteria. Among these patients, nearly 2% of them experienced early cardiac irAEs with concurrent hospitalizations. These patients had diverse sociodemographic backgrounds, unique comorbidity burdens, and varying levels of medication exposures. Using hundreds of features to represent these patient characteristics, we implemented a stepwise ML model building approach utilizing three different ML algorithms to evaluate the extent to which early cardiac irAEs can be predicted with baseline patient data. Our findings demonstrate the potential for ML models to identify patients at risk of these serious cardiac irAEs. Furthermore, they highlight potential risk factors for clinicians to consider in patient care and for future predictive risk models.

All three models had comparable predictive performance of cardiac irAEs, with AUC measures, *G*-means scores, and balanced accuracy falling within a modest range. Observed performance did not approach the higher thresholds typically considered necessary for clinical implementation and should therefore be interpreted as exploratory [36, 37]. Of note, positive predictive values below 5% for all models suggest significant progress is needed. However, these results are not entirely unexpected, as classification of rare events is very challenging even with ML approaches [38]. Our approach utilized data that is more routinely available, as opposed to depending on circulating biomarkers, labs, and cancer specific imaging, histology, and tissue testing reports, which may have provided more nuanced predictions. Several steps were taken to force models to positively classify rare outcomes in our methodology, which also influenced performance (i.e., downsampling strategies, *G*-means optimization) [39]. To address these challenges, others have emphasized broader classes of cardiac irAEs and have achieved better performance; however, this ambiguous criteria creates uncertainty as to whether positive cases are truly irAEs or reflect underlying comorbid conditions [40, 41]. In the study conducted by Heilbroner et al., utilizing a broader definition of cardiac adverse events including arrythmia and heart failure, had an adverse event rate of 8.4% among 4960 patients. [41] Their model predicting cardiac irAEs within 100 days from the index date achieved an AUC of 0.65, whereas the model in the present study achieved an AUC of 0.70. Differences in study design, cohort size, and outcome definitions may contribute to these variations. Larger sample sizes may allow future studies to define outcomes more precisely and further refine predictive models for clinical application.

Elastic net logistic regression and gradient boosted tree approaches demonstrated more nuanced separation of positive and negative classes when considering calibration curves. This reflects overfitting of the random forest model to the training data, as shown by the magnitude of the difference in predictive performance between training and testing data (AUC 0.928 versus 0.719). Exploration of different training approaches and feature sets may be warranted to better harness random forest algorithms. Furthermore, for elastic net and gradient boosted tree approaches, final models utilizing less than half of the initial feature set achieved comparable performance to initial models, demonstrating that future efforts can consider a more confined feature space for more practical approaches.

Our risk tier assignment strategy based on model outputs indicated that ML models could provide nuance into potential cardiac irAE risk. Most patients experiencing cardiac irAEs were classified as medium or high risk, and the high-risk tier had nearly 3 times the rate of adverse events compared to lower risk tiers for all models. This suggests a level of clinical translatability compatible with future refined modeling strategies. Patients can be triaged according to their assigned risk at baseline, where resources are allocated for increased follow-up visits and more rigorous monitoring including electrocardiograms, cardiac imaging, and blood biomarkers during ICI treatment [42, 43]. Early identification of cardiac irAEs prior to severe symptoms and hospitalizations may improve treatment outcomes [44, 45]. Our risk tiering approach was exploratory in nature to highlight patients at high risk; future endeavors can consider true costs of false positives versus the preventative benefit to better establish thresholds. For instance, if the cost of a false positive was relatively low, where “at-risk patients” were assigned to receive patient education initiatives or referrals to specialists, a looser classification threshold could be considered. However, if false positives lead to expensive tests or even hospital admissions, more stringent positive classification cut-offs would likely be necessary. These factors would further be balanced by the considered “cost” of ICI-induced cardiac irAEs, including finances, immediate health impact, and long-term implications of cancer treatment toxicities.

In evaluating key predictive features for ML models, we found cancer type plays a significant role in prediction of cardiac irAEs. Most prominently, patients with metastatic cancer appear to be at greater risk of cardiac irAEs along with patients with a primary lung cancer diagnosis, as both features significantly influenced model performance. Literature does suggest that primary cancer type can predispose patients to certain classes of irAEs, potentially due to tumor-specific effects on implicated organ systems or general severity of the illness [46]. Other literature suggests that different lung cancers significantly increase the likelihood of cardiac complications, even independent of therapeutic exposures [47]. Patients with metastatic cancer also face unique risk factors for cardiac complications, as significant metastases can cause systemic immune responses that worsen cardiac health [48–50]. Although we observed that cancer diagnosis type played a significant role in cardiac irAEs, characterizing cancer status from diagnostic codes limited analysis [51]. Future studies can utilize cohorts with more formal diagnoses based on histological reports and nuanced staging information to better characterize the influence of cancer subtypes and progression.

Our modeling efforts highlighted that different baseline comorbidities play significant roles in ML model predictions of cardiac irAEs. Most prominent among multiple models included weight loss, pulmonary circulation disorders, chronic pulmonary disease, cardiac arrhythmia, and fluid/electrolyte disorder. Having a history of arrhythmia may suggest suboptimal cardiovascular health for patients, making them more susceptible to cardiac irAEs when starting ICI therapy [52]. Pulmonary circulation disorders and chronic pulmonary disease have an immediate relationship with worsened cardiovascular function [53]. Diagnoses representative of weight loss and fluid/electrolyte disorders represent patients with significant nutritional deficiencies associated with cardiac complications [54]. While some of the key diagnoses may seem vague or simply representative of poor health, it is noteworthy that cumulative comorbidity index representations were not as significant across all models. Future efforts incorporating specific measurements of cardiovascular/pulmonary health and function, along measures of weight changes and nutritional status (electrolytes, vitamin deficiencies), may significantly improve risk assessment models.

Several different past medication exposures were also highlighted as key features in the predictive models. CNS suppressive medications were emphasized, including other cough suppressants (R05DB), opioids (N02A), and benzodiazepine derivatives (N05BA). CNS suppressive medications have been linked to an increased risk of several cardiovascular complications and recently have been shown to modulate immune activity and cell composition [55, 56]. Pain can represent cancer severity, and recent literature has suggested chronic pain can lead to immune dysregulation [57]. Furthermore, beta-blocking agents (C07) were meaningful predictors in multiple models, possibly representative of patients having pre-existing cardiovascular conditions that would increase their susceptibility to cardiac toxicities. Considering medication exposures as potential predictors does bring unique challenges, as it is difficult to distinguish whether the information serves is a surrogate for disease severity, symptom burden, or comorbidity or whether specific pharmacology leaves patients more disposed cardiac toxicities. Future studies can follow medication exposures over time to identify more robust associations.

While our study considered many different baseline patient characteristics, their influence on model predictions was heterogeneous. Index ICI therapy clearly did play a role, as combination ICI therapy was a highly ranked feature for multiple ML models, along with PD-1 and PD-L1 monotherapy. This is not unexpected, as multiple studies have shown that combination therapy increases the risk of many different irAEs, including myocarditis [52, 58]. Biological sex and race/ethnicity designations had some influence in predictive models, although their emphasis was mixed depending on the approach. Literature does suggest that men are predisposed to these events more than women and that White/Caucasians have a higher rate of cases relative to other ethnic backgrounds [58, 59]. None of the models seemed to strongly emphasize patient age; perhaps its role in the development of acute events is limited and age plays a greater role with the chronic onset of ICI-induced cardiac irAEs.

Our study design presents limitations to our findings. Removing patients with a prior history of cardiac inflammation or with ambiguous diagnoses does introduce a potential risk of sampling bias. However, it ensured identified cardiac irAEs were not attributable to pre-existing conditions or unclear diagnoses, creating a clearer outcome. While requiring concurrent hospitalization increased the specificity of the outcome, cardiac irAEs were still ascertained from diagnostic codeswithout biomarker, imaging, biopsy, or adjudication data possibly causing overestimate or missclassification of true immune-related cardiac toxicity. Such confirmatory data were not available in this dataset. Excluding patients with limited ICI exposure may also introduce selection bias, as early discontinuation can reflect other clinical events (e.g., other irAEs or disease progression) rather than limited reporting alone, which may limit generalizability to patients with short treatment courses. The absence of a diagnostic or medication code in the EHR was treated as absence of the condition, which may misclassify unrecorded exposures or diagnoses. Our study emphasized early onset cardiac irAEs due to its cross-sectional design, given that the health status of patients receiving cancer treatment can change rapidly over time. However, early-onset events may have distinct causes and implications relative to those occurring later, justifying this approach. Development of features that more explicitly represent polypharmacy, treatment intensity, and overlapping comorbid conditions might improve model performance beyond the independent variables considered. Future efforts can further explore refined feature selection strategies to embrace more nuanced resampling approaches as well (e.g., SMOTE, ROSE). Stepwise recursive feature elimination is one approach that can refine feature selection in a nuanced way and suggest characteristics vital for accurate predictions; however, with our broad feature set and fixed computational limits, we defer these experiments to future efforts.

Despite limitations, our study suggests ML approaches may provide needed help identifying patients at risk for serious ICI-induced cardiac irAEs. The growing population of patients receiving ICI agents and the emergence of large repositories of EHR data such as TriNetX will enable continued development of risk assessment tools for these events. Further studies can examine the most influential risk factors for cardiac irAEs to prioritize in future modeling efforts. Translational efforts creating meaningful risk assessment tools utilizing big data have the potential to significantly improve patient safety and optimize clinical outcomes with ICI treatment.

## Supplementary Information

Below is the link to the electronic supplementary material.

ESM 1 (DOCX 623 KB)

ESM 2 (DOCX 38.8 KB)

## Data Availability

The data underlying this study were collected within TriNetX electronic health records, by means of institutional access through the University of California Irvine. The TriNetX policy states their data can only be accessed “by researchers that are either part of the network or have a collaboration agreement with TriNetX.”

## Abbreviations

ICI: Immune checkpoint inhibitor
irAE: Immune-related adverse event
ML: Machine learning
AUC: Area under the curve
SHAP: Shapley additive explanations
