# Supplementary material for: Machine learning approaches to predict early cardiac immune-related adverse events in patients receiving immune checkpoint inhibitors
Source: Support Care Cancer. 2026 Jul 29;34(8):809. doi: 10.1007/s00520-026-10984-5 (PMC13415296; doi:10.1007/s00520-026-10984-5)
Supplement: Supplementary file 1 — (DOCX 623 KB) [file 520_2026_10984_MOESM1_ESM.docx]

**Supplemental Materials Index**

**Supplemental Figures**

| Supplemental Figure 1: | Retrospective Study Data Collection Strategy |
| --- | --- |
| Supplemental Figure 2: | Machine Learning Methods Implemented for All Modeling Strategies |
| Supplemental Figure 3: | Retrospective Study Population Creation |
| Supplemental Figure 4: | Histogram of Cardiac Immune-Related Adverse Event Timing within 90-Day Study Window |

**Supplemental Tables**

| Supplemental Table 1: | Diagnosis Codes Representing Cardiac Immune Related Adverse Events |
| --- | --- |
| Supplemental Table 2: | ML Models Utilized and Tuning Grids Utilized to Hyper-Parameter Grid Search |
| Supplemental Table 3: | Sampling and weighting Approaches Considered for Machine Learning Models |
| Supplemental Table 4: | Distribution of Patient Demographic Characteristics |
| Supplemental Table 5: | Distribution of Comorbid Conditions |
| Supplemental Table 6: | Distribution of Medication Exposures |
| Supplemental Table 7: | Highest G-means Values from Initial Sampling and Feature Weighting Evaluation |
| Supplemental Table 8: | Feature Importance Measures Utilized to for Feature Selection with Initial Model |
| Supplemental Table 9: | Model Performance Measures for Initial Models Using Training and Testing Data |
| Supplemental Table 10: | Feature Importance and Mean SHAP Values for all Features Included in Final Elastic Net Logistic Regression Model |
| Supplemental Table 11: | Feature Importance and Mean SHAP Values for all Features Included in Final Gradient Boosted Tree Model |
| Supplemental Table 12: | Feature Importance and Mean SHAP Values for all Features Included in Final Random Forest Model |
| Supplemental Table 13:  Supplemental Table 14: | Cardiac Immune Related Adverse Event Rate Across Ranked Quantiles Used for Model Calibration Curves  Top 20 Features by Feature Importance for Each ML Model |

**
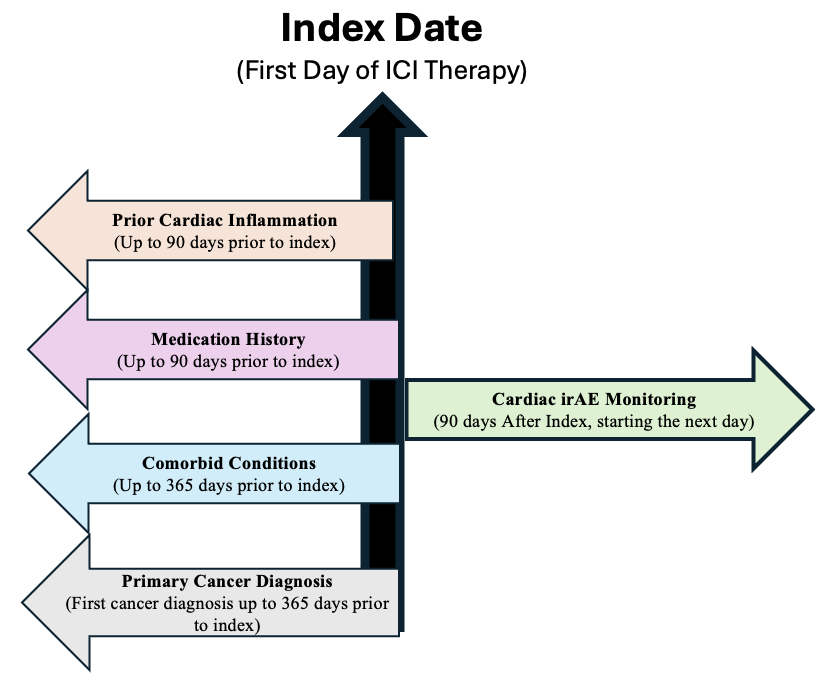
**

**Supplemental Figure 1: Retrospective Study Data Collection Strategy**

Study index date was date ICI therapy was initiated (Blackened arrow). All data collected was relative to this fixed timepoint with respect to retrospective study. Arrows pointing to the left represent data collection windows utilized for different features for predictive modeling, while the arrow pointing to the right represents the time frame cardiac irAE data was captured.

**
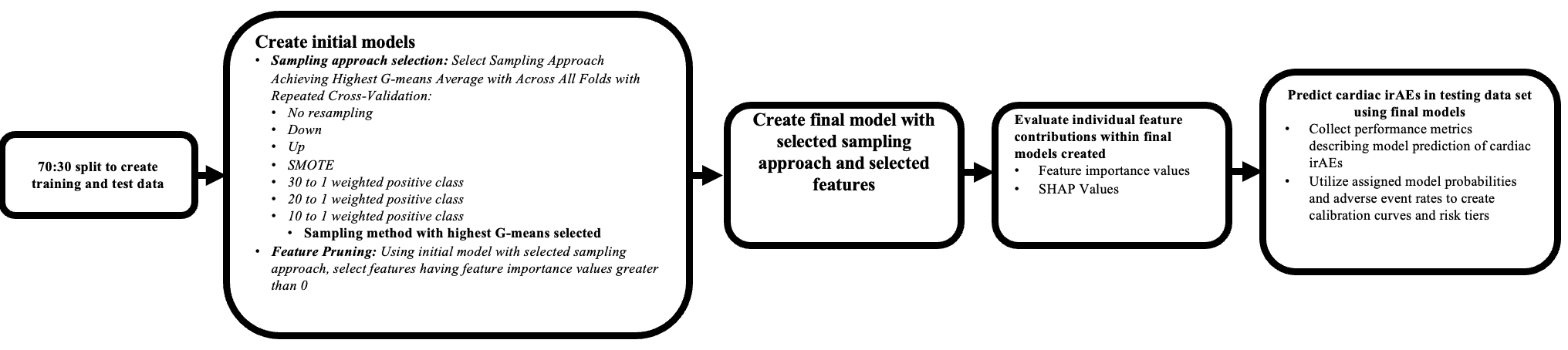
**

**Supplemental Figure 2: Machine Learning Methods Implemented for All Modeling Strategies**

This flow chart broadly summarizes machine learning steps implemented for each ML model starting with splitting the data set into training and testing data (far left), training ML models in subsequent steps, until predictions are made on the reserved testing data (far right).

**
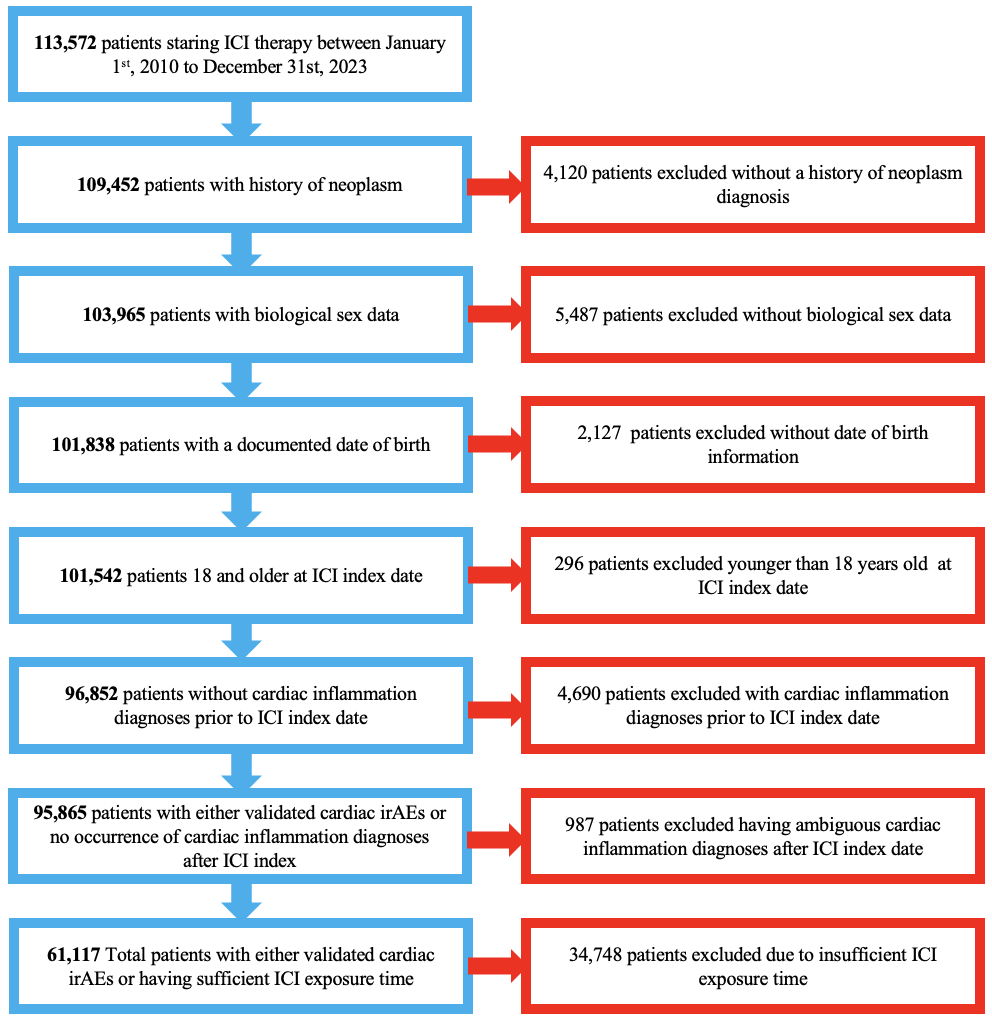
**

**Supplemental Figure 3: Retrospective Study Population Creation**

This is a flow chart showing study population creation, starting with data requisition from TriNetX at the top, followed by implementation of each inclusion/exclusion criteria selected shown with downward arrows. Each blue line pointing down indicates the implementation of the next criteria creating the patient population, with red lines to the right designating the number of patients removed due to the implementation of a given criteria.

**
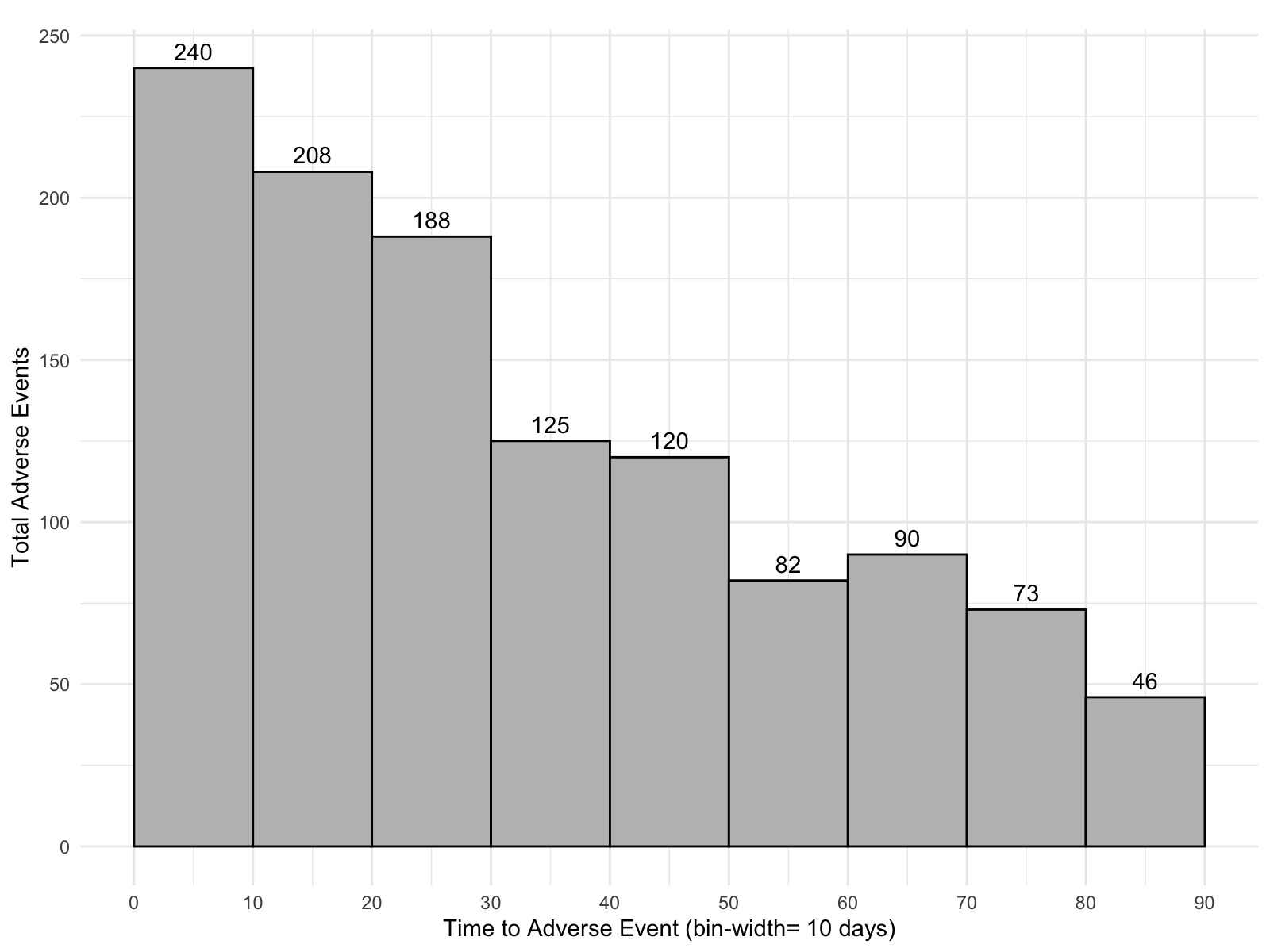
**

**Supplemental Figure 4:** Histogram of Cardiac Immune-Related Adverse Event Timing within 90-Day Study Window

The y-axis represents the count of total adverse events, with the x-axis representing bin-widths of 10 day increments up to 90 days from index date. The total number of patients represented in each bin is printed at the top of each bar in the plot.

**Supplemental Table 1: Diagnosis Codes Representing Cardiac Immune Related Adverse Events**

| ***Diagnosis*** | **ICD-10 Codes** | **ICD-9 Codes** |
| --- | --- | --- |
| *Myocarditis* | I41.1, I41.8, I41.9, I51.4, I41 | 422.91, 422.93, 422.99, 422.90, 422, 429.0, 422.0 |
| *Pericarditis* | I30.0, I30.8, I30.9, I32 | 420.91, 420.99, 420, 420.9, 420.90, 115.03, 115.13, 115.93, 420.0 |
| *Pericardial Disease* | I31.2, I31.3, I31.4, I31.8, I31.9 | 423.0, 423.3, 423.8, 423.9, 422.0 |

**Supplemental Table 2: ML Models Utilized and Tuning Grids Utilized to Hyper-Parameter Grid Search**

From left to right, the first column indicates the machine learning approach utilized, followed by a basic description of it along with its hyper-parameters utilized in model training. The last column indicates hyper-parameter values considered within tuning grids for each given model within their perspective tuning grids.

| ***Model Type*** | **Description** | **Tuning Grid** |
| --- | --- | --- |
| *Elastic Net Logistic Regression* | A generalized logistic regression algorithm with elastic net regularization. **Lambda** controls the extent of “shrinkage” to coefficients, higher lambdas lead to more coefficient reduction. **Alpha** controls the balance of lasso and ridge regression approaches, with values close to 1 associated with Lasso regression approaches and those close to 0 associated with ridge regression behavior. | **Lambda Values:** 1.00e-06, 2.07e-06, 4.28e-06, 8.86e-06, 1.83e-05, 3.79e-05, 7.85e-05, 1.62e-04, 3.36e-04, 6.95e-04, 1.44e-03, 2.98e-03, 6.16e-03, 1.27e-02, 2.64e-02, 5.46e-02, 1.13e-01, 2.34e-01, 4.83e-01, 1.00e+00  **Alpha Values:** 0.1, 0.2, 0.3, 0.4, 0.5, 0.6, 0.7, 0.8, 0.9 |
| *Gradient Boosted Trees* | A gradient boosted algorithm that builds an ensemble of decision trees. **Max Depth** controls the “depth” of individual decision trees created. **Learning Rate (eta)** controls the extent created trees impact subsequent builds. **Minimum child weight** defines the relevant sample needed in a decision trees in order to create a new “leaf”. The number of rounds utilized (100 total) was fixed due to computational constraints. | **Max Depth** = 2, 3, 4, 5  **Learning Rate=** 0.05, 0.1, 0.25, 0.5  **Minimum Child Weight** = 1, 5, 10, 20, 30, 40, 50 |
| *Random Forest* | A more efficient version of the random forest algorithm. “**MTRY**”, controls the number of variables attempted for each tree split. **Minimum Node Size** defines the number of samples needed in a terminal node. | **mtry** = 4, 8, 12, 16, 24, 32, 40, 48  **Minimum Node Size** = 1, 5, 10, 20, 30, 40, 50 |

**Supplemental Table 3: Sampling and weighting Approaches Considered for Machine Learning Models**

Each row represents a re-sampling or weighting approach considered for machine learning, followed by a basic description of how they work.

| ***Sampling Approach*** | **Description** |
| --- | --- |
| *No Resampling* | No specific resampling approach implemented, just three-fold cross validation of all entries in the data |
| *Down* | Within each created fold, reduces class imbalance by randomly removing majority class observations until both classes are equal in size |
| *Up* | Within each created fold, reduces class imbalance by increasing the size of the minority class through random duplication of existing observations until both classes are balanced. |
| *SMOTE* | Within each created fold, reduces class imbalance by creating synthetic examples of the minority class by interpolating between existing observations. |
| *Rose* | Within each created fold, reduces class imbalance by creating synthetic examples of the minority class with different approaches than SMOTE, including boot strap sampling and kernel density estimation. |
| *30 to 1 weighted* | Instead of resampling, this approach applies a weight to emphasize the minority class. With a 30:1 weight ratio, each positive response is treated as if it were 30 entries, while each negative response is treated as a single entry. This increases the model's sensitivity to the minority class without altering the dataset size. |
| *20 to 1 weighted* | Instead of resampling, this approach applies a weight to emphasize the minority class. With a 20:1 weight ratio, each positive response is treated as if it were 20 entries, while each negative response is treated as a single entry. This increases the model's sensitivity to the minority class without altering the dataset size. |
| *10 to 1 weighted* | Instead of resampling, this approach applies a weight to emphasize the minority class. With a 10:1 weight ratio, each positive response is treated as if it were 10 entries, while each negative response is treated as a single entry. This increases the model's sensitivity to the minority class without altering the dataset size. |

**Supplemental Table 4: Distribution of Patient Demographic Characteristics**

From left to right, the first column represents feature names. The percentage of each feature is reported, followed by the total count below amongst patients experiencing cardiac irAEs (Cardiac irAE group), not experiencing cardiac irAEs (No- Cardiac irAE Group), amongst the entire patient population (All Patients), Training and Testing data. Blank entries in Train and Test data columns indicate features were not selected modeling efforts due to lack of prevalence amongst patients experiencing cardiac irAEs.

| **Feature** | **All Patients** | **No irAE** | **irAE** | **Train** | **Test** |
| --- | --- | --- | --- | --- | --- |
| *Age 45 to 64* | 37.4 (22859) | 37.4 (22419) | 37.5 (440) | 37.3 (15965) | 37.6 (6894) |
| *Age 65 to 74* | 33.2 (20311) | 33.3 (19942) | 31.5 (369) | 33.4 (14270) | 32.9 (6041) |
| *Age 75 up* | 21.8 (13321) | 21.8 (13053) | 22.9 (268) | 21.7 (9274) | 22.1 (4047) |
| *Male* | 56.7 (34637) | 56.7 (33992) | 55 (645) | 56.3 (24092) | 57.5 (10545) |
| *White* | 72.6 (44345) | 72.5 (43468) | 74.8 (877) | 72.5 (31013) | 72.7 (13332) |
| *Black* | 9 (5491) | 8.9 (5350) | 12 (141) | 9.1 (3877) | 8.8 (1614) |
| *Hispanic* | 4.5 (2746) | 4.5 (2693) | 4.5 (53) | 4.4 (1888) | 4.7 (858) |
| *Asian* | 4.2 (2590) | 4.3 (2551) | 3.3 (39) | 4.2 (1793) | 4.3 (797) |
| *PD-1 Index* | 73.3 (44797) | 73.3 (43926) | 74.3 (871) | 73.3 (31365) | 73.3 (13432) |
| *PDL-1 Index* | 17.1 (10476) | 17.2 (10308) | 14.3 (168) | 17.1 (7330) | 17.2 (3146) |
| *CTLA-4 Index* | 2.5 (1531) | 2.5 (1520) | 0.9 (11) | 2.5 (1073) | 2.5 (458) |
| *Combination Therapy Index* | 7.1 (4313) | 7 (4191) | 10.4 (122) | 7 (3014) | 7.1 (1299) |
| *Medium NCI Score* | 34.2 (20881) | 34.2 (20518) | 31 (363) | 34.2 (14617) | 34.2 (6264) |
| *High NCI Score* | 30.6 (18729) | 30.3 (18152) | 49.2 (577) | 30.6 (13071) | 30.9 (5658) |
| *Lung Cancer* | 26.2 (15986) | 25.9 (15544) | 37.7 (442) | 26.1 (11173) | 26.3 (4813) |
| *Skin Cancer* | 15.7 (9595) | 15.8 (9489) | 9 (106) | 15.7 (6696) | 15.8 (2899) |
| *Other Digestive Cancer* | 5.4 (3287) | 5.4 (3213) | 6.3 (74) | 5.3 (2277) | 5.5 (1010) |
| *Renal Cancer* | 7.3 (4478) | 7.4 (4407) | 6.1 (71) | 7.3 (3119) | 7.4 (1359) |
| *Breast Cancer* | 5.3 (3256) | 5.4 (3210) | 3.9 (46) | 5.4 (2323) | 5.1 (933) |
| *Oral Cancer* | 4.1 (2509) | 4.1 (2466) | 3.7 (43) | 4.1 (1755) | 4.1 (754) |
| *Bladder Cancer* | 4.1 (2503) | 4.1 (2460) | 3.7 (43) | 4 (1719) | 4.3 (784) |
| *Blood Cancer* | 2.6 (1619) | 2.6 (1581) | 3.2 (38) | 2.7 (1145) | 2.6 (474) |
| *Other Respiratory Cancer* | 1.6 (967) | 1.6 (934) | 2.8 (33) | 1.6 (700) | 1.5 (267) |
| *Liver Cancer* | 4.2 (2589) | 4.3 (2558) | 2.6 (31) | 4.1 (1763) | 4.5 (826) |
| *Mesothelial Soft Tissue Cancer* | 1.7 (1068) | 1.7 (1038) | 2.6 (30) | 1.7 (748) | 1.7 (320) |
| *Colorectal Cancer* | 2.3 (1380) | 2.3 (1356) | 2 (24) | 2.3 (985) | 2.2 (395) |
| *Central Nervous System Cancer* | 1.3 (811) | 1.3 (791) | 1.7 (20) | 1.4 (584) | 1.2 (227) |
| *Uterine Cancer* | 2.4 (1476) | 2.4 (1461) | 1.3 (15) |  |  |
| *Prostate Cancer* | 1.9 (1135) | 1.9 (1122) | 1.1 (13) |  |  |
| *Ovarian Cancer* | 0.8 (475) | 0.8 (462) | 1.1 (13) |  |  |
| *Cervical Cancer* | 1.1 (690) | 1.1 (681) | 0.8 (9) |  |  |
| *Other Genitourinary Cancer* | 0.9 (539) | 0.9 (532) | 0.6 (7) |  |  |
| *Female Other Cancer* | 0.5 (319) | 0.5 (313) | 0.5 (6) |  |  |
| *Bone Cancer* | 0.4 (228) | 0.4 (224) | 0.3 (4) |  |  |
| *Endocrine Cancer* | 0.5 (335) | 0.6 (333) | 0.2 (2) |  |  |

**Supplemental Table 5: Distribution of Comorbid Conditions**

From left to right, the first column represents feature names. The percentage of each feature is reported, followed by the total count below amongst patients experiencing cardiac irAEs (Cardiac irAE group), not experiencing cardiac irAEs (No- Cardiac irAE Group), amongst the entire patient population (All Patients), Training and Testing data. Blank entries in Train and Test data columns indicate features were not selected modeling efforts due to lack of prevalence amongst patients experiencing cardiac irAEs.

| **Condition** | **All Patients** | **No irAE** | **irAE** | **Train** | **Test** |
| --- | --- | --- | --- | --- | --- |
| *Metastatic Cancer* | 67.8 (41446) | 67.5 (40441) | 85.8 (1005) | 67.5 (28873) | 68.6 (12573) |
| *Cardiac Arrythmia* | 23.7 (14506) | 23.3 (13978) | 45.1 (528) | 23.7 (10139) | 23.8 (4367) |
| *Uncomplicated Hypertension* | 37.1 (22676) | 37 (22152) | 44.7 (524) | 37.1 (15852) | 37.2 (6824) |
| *Fluid/Electrolyte Disorder* | 22 (13463) | 21.6 (12975) | 41.6 (488) | 22.2 (9505) | 21.6 (3958) |
| *Weight Loss* | 17.3 (10554) | 16.8 (10083) | 40.2 (471) | 17.1 (7331) | 17.6 (3223) |
| *Chronic Pulmonary Disease* | 24.5 (14954) | 24.2 (14495) | 39.2 (459) | 24.5 (10489) | 24.4 (4465) |
| *Depression* | 15.6 (9533) | 15.4 (9220) | 26.7 (313) | 15.7 (6726) | 15.3 (2807) |
| *Liver Disease* | 18.3 (11157) | 18.1 (10853) | 25.9 (304) | 18.2 (7779) | 18.4 (3378) |
| *Peripheral Vascular Diseases* | 15 (9188) | 14.9 (8915) | 23.3 (273) | 15.1 (6439) | 15 (2749) |
| *Neurological Disorders* | 9.5 (5790) | 9.2 (5526) | 22.5 (264) | 9.3 (3992) | 9.8 (1798) |
| *Coagulopathy* | 10.7 (6538) | 10.5 (6293) | 20.9 (245) | 10.6 (4524) | 11 (2014) |
| *Renal Failure* | 12.3 (7498) | 12.1 (7271) | 19.4 (227) | 12.3 (5255) | 12.2 (2243) |
| *Obesity* | 13.5 (8259) | 13.4 (8038) | 18.9 (221) | 13.3 (5702) | 13.9 (2557) |
| *Hypothyroidism* | 13.9 (8485) | 13.8 (8282) | 17.3 (203) | 13.9 (5953) | 13.8 (2532) |
| *Congestive Heart Failure* | 7.8 (4757) | 7.6 (4561) | 16.7 (196) | 7.8 (3318) | 7.8 (1439) |
| *Pulmonary Circulation Disorders* | 7.2 (4375) | 7 (4187) | 16 (188) | 7.2 (3079) | 7.1 (1296) |
| *Drug Abuse* | 6.6 (4025) | 6.4 (3847) | 15.2 (178) | 6.6 (2841) | 6.5 (1184) |
| *Uncomplicated Diabetes* | 9.9 (6081) | 9.8 (5904) | 15.1 (177) | 10 (4258) | 9.9 (1823) |
| *Complicated Hypertension* | 10.1 (6196) | 10 (6021) | 14.9 (175) | 10 (4293) | 10.4 (1903) |
| *Valvular Disease* | 7.8 (4747) | 7.7 (4588) | 13.6 (159) | 7.9 (3370) | 7.5 (1377) |
| *Deficiency Anemia* | 7.5 (4583) | 7.4 (4449) | 11.4 (134) | 7.5 (3202) | 7.5 (1381) |
| *Complicated Diabetes* | 9.4 (5724) | 9.3 (5590) | 11.4 (134) | 9.3 (3988) | 9.5 (1736) |
| *Alcohol Abuse* | 5.5 (3342) | 5.4 (3222) | 10.2 (120) | 5.5 (2334) | 5.5 (1008) |
| *Rheumatoid Arthritis/ Colligated Vascular Disease* | 3.9 (2379) | 3.8 (2275) | 8.9 (104) | 3.9 (1673) | 3.9 (706) |
| *Paralysis* | 2.4 (1473) | 2.3 (1387) | 7.3 (86) | 2.4 (1039) | 2.4 (434) |
| *Acute Myocardial Infarction* | 2.6 (1585) | 2.5 (1509) | 6.5 (76) | 2.6 (1092) | 2.7 (493) |
| *Peptic Ulcer Disease* | 2.2 (1342) | 2.1 (1268) | 6.3 (74) | 2.2 (930) | 2.2 (412) |
| *History of Myocardial Infarction* | 3.8 (2352) | 3.8 (2290) | 5.3 (62) | 3.9 (1672) | 3.7 (680) |
| *Blood Loss Anemia* | 2.7 (1655) | 2.7 (1608) | 4 (47) | 2.7 (1134) | 2.8 (521) |
| *AIDs* | 0.8 (500) | 0.8 (471) | 2.5 (29) | 0.8 (360) | 0.8 (140) |
| *Psychosis* | 0.9 (541) | 0.9 (516) | 2.1 (25) | 0.9 (374) | 0.9 (167) |

**Supplemental Table 6: Distribution of Medication Exposures**

From left to right, the first column represents feature names. The percentage of each feature is reported, followed by the total count below amongst patients experiencing cardiac irAEs (Cardiac irAE group), not experiencing cardiac irAEs (No- Cardiac irAE Group), amongst the entire patient population (All Patients), Training and Testing data. Blank entries for Training and Testing data indicate that the feature wasn’t included in modeling exercises. Blank entries in Train and Test data columns indicate features were not selected modeling efforts due to lack of prevalence amongst patients experiencing cardiac irAEs.

| **Medications** | **All Patients** | **No irAE** | **irAE** | **Train** | **Test** |
| --- | --- | --- | --- | --- | --- |
| *opioids (n02a)* | 63 (38506) | 62.8 (37621) | 75.5 (885) | 62.9 (26923) | 63.2 (11583) |
| *serotonin (5ht3) antagonists (a04aa)* | 66.4 (40562) | 66.2 (39689) | 74.5 (873) | 66.5 (28463) | 66 (12099) |
| *corticosteroids, dermatological preparations (d07)* | 66 (40360) | 65.9 (39516) | 72 (844) | 66.1 (28285) | 65.9 (12075) |
| *corticosteroids for systemic use, plain (h02a)* | 65.4 (39982) | 65.3 (39147) | 71.2 (835) | 65.6 (28045) | 65.1 (11937) |
| *anilides (n02be)* | 52 (31764) | 51.6 (30961) | 68.5 (803) | 52 (22259) | 51.8 (9505) |
| *antiarrhythmics, class i and iii (c01b)* | 48.6 (29730) | 48.4 (29029) | 59.8 (701) | 48.6 (20808) | 48.7 (8922) |
| *heparin group (b01ab)* | 48.2 (29465) | 48 (28785) | 58 (680) | 48.4 (20692) | 47.8 (8773) |
| *opioid anesthetics (n01ah)* | 45.2 (27616) | 45 (26975) | 54.7 (641) | 45.1 (19280) | 45.5 (8336) |
| *antihistamines for systemic use (r06a)* | 51.2 (31282) | 51.1 (30654) | 53.6 (628) | 51.1 (21878) | 51.3 (9404) |
| *drugs for constipation (a06a)* | 37.5 (22923) | 37.2 (22317) | 51.7 (606) | 37.5 (16043) | 37.5 (6880) |
| *potassium (a12b)* | 35.5 (21682) | 35.3 (21147) | 45.6 (535) | 35.5 (15195) | 35.4 (6487) |
| *antiinfectives and antiseptics, excl. combinations with corticosteroids (g01a)* | 36.7 (22404) | 36.5 (21882) | 44.5 (522) | 36.8 (15724) | 36.4 (6680) |
| *benzodiazepine derivatives (n05cd)* | 35.4 (21647) | 35.3 (21142) | 43.1 (505) | 35.2 (15074) | 35.8 (6573) |
| *cardiac stimulants excl. cardiac glycosides (c01c)* | 38 (23233) | 38 (22753) | 41 (480) | 37.9 (16221) | 38.2 (7012) |
| *platinum compounds (l01xa)* | 34.5 (21091) | 34.4 (20620) | 40.2 (471) | 34.4 (14710) | 34.8 (6381) |
| *phenothiazines (n05a_c)* | 31.8 (19448) | 31.7 (18978) | 40.1 (470) | 32.1 (13715) | 31.3 (5733) |
| *proton pump inhibitors (a02bc)* | 25 (15266) | 24.7 (14814) | 38.6 (452) | 25 (10684) | 25 (4582) |
| *beta blocking agents (c07)* | 23.6 (14416) | 23.3 (13988) | 36.5 (428) | 23.4 (10000) | 24.1 (4416) |
| *calcium (a12a)* | 28.2 (17235) | 28 (16809) | 36.3 (426) | 28.1 (12033) | 28.4 (5202) |
| *cephalosporins 1_4 (j01db_e)* | 29 (17698) | 28.8 (17287) | 35.1 (411) | 28.8 (12319) | 29.3 (5379) |
| *benzodiazepine derivatives (n05ba)* | 24.9 (15241) | 24.7 (14834) | 34.7 (407) | 24.8 (10613) | 25.2 (4628) |
| *opium alkaloids and derivatives (r05da)* | 21.6 (13190) | 21.3 (12786) | 34.5 (404) | 21.8 (9323) | 21.1 (3867) |
| *other general anesthetics (n01ax)* | 29.3 (17884) | 29.2 (17481) | 34.4 (403) | 29.1 (12460) | 29.6 (5424) |
| *vitamin k and other hemostatics (b02b)* | 32.1 (19612) | 32.1 (19226) | 32.9 (386) | 32 (13683) | 32.3 (5929) |
| *intestinal antiinflammatory agents (a07e)* | 26.3 (16080) | 26.2 (15699) | 32.5 (381) | 26.3 (11271) | 26.2 (4809) |
| *drugs for constipation (a06aa)* | 18 (11012) | 17.8 (10651) | 30.8 (361) | 18.1 (7724) | 17.9 (3288) |
| *acidifiers (g04ba)* | 24 (14646) | 23.8 (14292) | 30.2 (354) | 23.9 (10236) | 24.1 (4410) |
| *hmg coa reductase inhibitors (c10aa)* | 20.9 (12774) | 20.7 (12423) | 29.9 (351) | 20.9 (8952) | 20.8 (3822) |
| *antimetabolites (l01b)* | 19.9 (12174) | 19.8 (11844) | 28.2 (330) | 19.7 (8446) | 20.3 (3728) |
| *plant alkaloids and other natural products (l01c)* | 22.8 (13941) | 22.7 (13624) | 27 (317) | 22.8 (9773) | 22.7 (4168) |
| *platelet aggregation agents (b01ac)* | 14.9 (9076) | 14.6 (8766) | 26.5 (310) | 14.8 (6320) | 15 (2756) |
| *other antiepileptics (n03ax)* | 18.9 (11546) | 18.7 (11237) | 26.4 (309) | 18.9 (8094) | 18.8 (3452) |
| *agents acting on the renin-angiotensin system (c09)* | 18.7 (11456) | 18.6 (11155) | 25.7 (301) | 18.8 (8056) | 18.5 (3400) |
| *salicylic acid and derivatives (n02ba)* | 14.2 (8662) | 13.9 (8361) | 25.7 (301) | 14.1 (6025) | 14.4 (2637) |
| *vitamin b12 and folic acid (b03b)* | 15.8 (9663) | 15.6 (9365) | 25.4 (298) | 15.8 (6752) | 15.9 (2911) |
| *calcium channel blockers (c08)* | 14.3 (8730) | 14.1 (8468) | 22.4 (262) | 14.3 (6099) | 14.3 (2631) |
| *other antibacterials (j01x)* | 14.4 (8784) | 14.2 (8525) | 22.1 (259) | 14.6 (6226) | 14 (2558) |
| *muscle relaxants, peripherally acting agents (m03a)* | 18.4 (11255) | 18.3 (10997) | 22 (258) | 18.4 (7886) | 18.4 (3369) |
| *beta-lactam antibacterials, penicillins (j01c)* | 11.1 (6779) | 10.9 (6525) | 21.7 (254) | 11.2 (4784) | 10.9 (1995) |
| *intestinal antiinfectives (a07a)* | 11.3 (6926) | 11.1 (6676) | 21.3 (250) | 11.4 (4896) | 11.1 (2030) |
| *high-ceiling diuretics (c03c)* | 11.4 (6995) | 11.3 (6745) | 21.3 (250) | 11.4 (4897) | 11.4 (2098) |
| *gabapentinoids (n02bf)* | 15.1 (9234) | 15 (8984) | 21.3 (250) | 15.2 (6495) | 14.9 (2739) |
| *propionic acid derviatives (m01ae)* | 12.1 (7407) | 12 (7187) | 18.8 (220) | 12 (5150) | 12.3 (2257) |
| *other gynecologicals (g02c)* | 12 (7338) | 11.9 (7119) | 18.7 (219) | 11.9 (5102) | 12.2 (2236) |
| *nasal decongestants for systemic use (r01b)* | 13.7 (8345) | 13.6 (8129) | 18.4 (216) | 13.7 (5881) | 13.4 (2464) |
| *insulins and analogues (a10a)* | 11.1 (6772) | 10.9 (6561) | 18 (211) | 11.1 (4728) | 11.1 (2044) |
| *vitamin a and d, incl. combinations of the two (a11c)* | 11.1 (6775) | 11 (6564) | 18 (211) | 11.1 (4736) | 11.1 (2039) |
| *other antidepressants (n06ax)* | 11.4 (6959) | 11.3 (6753) | 17.6 (206) | 11.4 (4895) | 11.3 (2064) |
| *other cardiac preparations (c01e)* | 11.3 (6930) | 11.2 (6728) | 17.2 (202) | 11.4 (4859) | 11.3 (2071) |
| *macrolides, lincosamides and streptogramins (j01f)* | 8.9 (5419) | 8.7 (5221) | 16.9 (198) | 8.9 (3815) | 8.7 (1604) |
| *muscle relaxants, centrally acting agents (m03b)* | 8.9 (5465) | 8.8 (5283) | 15.5 (182) | 9 (3855) | 8.8 (1610) |
| *direct factor xa inhibitors (b01af)* | 8 (4885) | 7.9 (4716) | 14.4 (169) | 8.1 (3456) | 7.8 (1429) |
| *expectorants (r05ca)* | 6.6 (4032) | 6.5 (3867) | 14.1 (165) | 6.5 (2795) | 6.7 (1237) |
| *thyroid preparations (h03a)* | 9.3 (5684) | 9.2 (5524) | 13.7 (160) | 9.3 (3995) | 9.2 (1689) |
| *melatonin receptor agonists (n05ch)* | 8 (4866) | 7.9 (4708) | 13.5 (158) | 8 (3402) | 8 (1464) |
| *low-ceiling diuretics, thiazides (c03a)* | 8.9 (5456) | 8.8 (5301) | 13.2 (155) | 9 (3868) | 8.7 (1588) |
| *antiseptics (r02aa)* | 11.3 (6892) | 11.2 (6741) | 12.9 (151) | 11.4 (4859) | 11.1 (2033) |
| *acetic acid derivatives (m01ab)* | 10.4 (6339) | 10.3 (6190) | 12.7 (149) | 10.5 (4493) | 10.1 (1846) |
| *other urologicals (g04bx)* | 7.3 (4439) | 7.2 (4294) | 12.4 (145) | 7.2 (3064) | 7.5 (1375) |
| *other cough suppressants (r05db)* | 4.7 (2890) | 4.6 (2749) | 12 (141) | 4.7 (2031) | 4.7 (859) |
| *selective serotonin reuptake inhibitors (n06ab)* | 8.3 (5102) | 8.3 (4966) | 11.6 (136) | 8.3 (3564) | 8.4 (1538) |
| *protein kinase inhibitors (l01e)* | 7.8 (4791) | 7.8 (4675) | 9.9 (116) | 7.8 (3334) | 7.9 (1457) |
| *glycogenolytic hormones (h04a)* | 6.6 (4021) | 6.5 (3910) | 9.5 (111) | 6.6 (2824) | 6.5 (1197) |
| *immunostimulants (l03a)* | 7.7 (4702) | 7.7 (4593) | 9.3 (109) | 7.7 (3282) | 7.7 (1420) |
| *alpha adrenoreceptor antagonists (g04ca)* | 6.5 (3984) | 6.5 (3880) | 8.9 (104) | 6.6 (2814) | 6.4 (1170) |
| *drugs used in nicotine dependence (n07ba)* | 4.5 (2725) | 4.4 (2622) | 8.8 (103) | 4.5 (1920) | 4.4 (805) |
| *hydrazinophthalazine derivatives (c02db)* | 6.9 (4240) | 6.9 (4140) | 8.5 (100) | 6.8 (2929) | 7.2 (1311) |
| *iron preparations (b03a)* | 4.7 (2863) | 4.6 (2765) | 8.4 (98) | 4.7 (1995) | 4.7 (868) |
| *tetracyclines (j01a)* | 4.1 (2525) | 4.1 (2430) | 8.1 (95) | 4.2 (1788) | 4 (737) |
| *antithrombotic enzymes (b01ad)* | 9.9 (6068) | 10 (5975) | 7.9 (93) | 10 (4268) | 9.8 (1800) |
| *sulfonamides and trimethoprim (j01e)* | 4.8 (2916) | 4.7 (2823) | 7.9 (93) | 4.7 (2024) | 4.9 (892) |
| *propulsives (a03f)* | 6.9 (4203) | 6.9 (4111) | 7.8 (92) | 6.9 (2932) | 6.9 (1271) |
| *antipropulsives (a07d)* | 4.8 (2946) | 4.8 (2856) | 7.7 (90) | 4.8 (2048) | 4.9 (898) |
| *other antiemetics (a04ad)* | 9.4 (5725) | 9.4 (5637) | 7.5 (88) | 9.3 (3980) | 9.5 (1745) |
| *belladonna and derivatives, plain (a03b)* | 3.9 (2394) | 3.9 (2316) | 6.7 (78) | 3.9 (1681) | 3.9 (713) |
| *biguanides (a10ba)* | 5.7 (3493) | 5.7 (3415) | 6.7 (78) | 5.7 (2420) | 5.9 (1073) |
| *ascorbic acid (vitamin c), incl. combinations (a11g)* | 4.4 (2700) | 4.4 (2624) | 6.5 (76) | 4.5 (1913) | 4.3 (787) |
| *agents against amoebiasis and other protozoan diseases (p01a)* | 4 (2475) | 4 (2402) | 6.2 (73) | 4.1 (1764) | 3.9 (711) |
| *benzodiazepine related drugs (n05cf)* | 3.9 (2403) | 3.9 (2331) | 6.1 (72) | 3.9 (1678) | 4 (725) |
| *antibiotics (r02ab)* | 5.6 (3440) | 5.6 (3368) | 6.1 (72) | 5.7 (2440) | 5.5 (1000) |
| *diazepines, oxazepines, thiazepines and oxepines (n05ah)* | 4.6 (2803) | 4.6 (2733) | 6 (70) | 4.6 (1953) | 4.6 (850) |
| *direct acting antivirals (j05a)* | 4.1 (2495) | 4 (2426) | 5.9 (69) | 4.1 (1747) | 4.1 (748) |
| *vitamin k antagonist (b01aa)* | 1.8 (1084) | 1.7 (1017) | 5.7 (67) | 1.8 (755) | 1.8 (329) |
| *vasodilators used in cardiac diseases (c01d)* | 3.3 (2045) | 3.3 (1978) | 5.7 (67) | 3.3 (1432) | 3.3 (613) |
| *triazole and tetrazole derivatives (j02ac)* | 3 (1816) | 2.9 (1750) | 5.6 (66) | 3 (1278) | 2.9 (538) |
| *uric acid production prevention (m04aa)* | 3.1 (1878) | 3 (1816) | 5.3 (62) | 3.1 (1309) | 3.1 (569) |
| *other drugs affecting bone structure and mineralization (m05bx)* | 3.1 (1888) | 3 (1826) | 5.3 (62) | 3.1 (1314) | 3.1 (574) |
| *aldosterone antagonists and other potassium-sparing agents (c03d)* | 2.7 (1626) | 2.6 (1567) | 5 (59) | 2.6 (1129) | 2.7 (497) |
| *bisphosphonates (m05ba)* | 3.4 (2059) | 3.3 (2000) | 5 (59) | 3.4 (1461) | 3.3 (598) |
| *other hypnotics and sedatives (n05cm)* | 5.1 (3109) | 5.1 (3050) | 5 (59) | 5.1 (2177) | 5.1 (932) |
| *urniary frequency and incontinence (g04bd)* | 2.8 (1714) | 2.8 (1657) | 4.9 (57) | 2.8 (1208) | 2.8 (506) |
| *parasympathomimetics (n07a)* | 4.6 (2828) | 4.6 (2772) | 4.8 (56) | 4.7 (2009) | 4.5 (819) |
| *halogenated hydrocarbons (n01ab)* | 1.7 (1057) | 1.7 (1002) | 4.7 (55) | 1.7 (736) | 1.8 (321) |
| *diphenylmethane derivatives (n05bb)* | 2.5 (1525) | 2.5 (1470) | 4.7 (55) | 2.5 (1053) | 2.6 (472) |
| *sex hormones (g03a_g)* | 3.3 (2024) | 3.3 (1971) | 4.5 (53) | 3.3 (1428) | 3.3 (596) |
| *aminoglycoside antibacterials (j01g)* | 3.2 (1952) | 3.2 (1900) | 4.4 (52) | 3.3 (1395) | 3 (557) |
| *vitamin b1, plain and in combination with vitamin b6 and b14 (a11d)* | 2.5 (1502) | 2.4 (1454) | 4.1 (48) | 2.5 (1058) | 2.4 (444) |
| *vitamin b1, plain and in combination with vitamin b6 and b13 (a11d)* | 2.5 (1502) | 2.4 (1454) | 4.1 (48) | 2.5 (1058) | 2.4 (444) |
| *vitamin b1, plain and in combination with vitamin b6 and b12 (a11d)* | 2.5 (1502) | 2.4 (1454) | 4.1 (48) | 2.5 (1058) | 2.4 (444) |
| *immunosuppressants (l04a)* | 1.9 (1144) | 1.8 (1096) | 4.1 (48) | 1.9 (810) | 1.8 (334) |
| *cancer endocrine therapies (l02)* | 3.8 (2316) | 3.8 (2269) | 4 (47) | 3.8 (1638) | 3.7 (678) |
| *other drugs for peptic ulcer and gastro-oesophageal reflux disease (gord) (a02bx)* | 2.5 (1555) | 2.5 (1513) | 3.6 (42) | 2.6 (1093) | 2.5 (462) |
| *alkylating agents (l01a)* | 2.5 (1533) | 2.5 (1491) | 3.6 (42) | 2.5 (1074) | 2.5 (459) |
| *other antineoplastic agents (l01xx)* | 2.9 (1799) | 2.9 (1758) | 3.5 (41) | 3 (1270) | 2.9 (529) |
| *butyrophenone derivatives (n05ad)* | 4 (2430) | 4 (2389) | 3.5 (41) | 4 (1700) | 4 (730) |
| *mucolytics (r05cb)* | 3.3 (2044) | 3.3 (2004) | 3.4 (40) | 3.4 (1448) | 3.3 (596) |
| *sulfonylureas (a10bb)* | 2.3 (1383) | 2.3 (1349) | 2.9 (34) | 2.2 (960) | 2.3 (423) |
| *xanthine derivatives (n06bc)* | 1.2 (726) | 1.2 (692) | 2.9 (34) | 1.2 (532) | 1.1 (194) |
| *erectile dysfunction drugs (g04be)* | 1.7 (1010) | 1.6 (977) | 2.8 (33) | 1.7 (720) | 1.6 (290) |
| *bacterial vaccines (j07a)* | 0.6 (363) | 0.6 (332) | 2.6 (31) | 0.6 (240) | 0.7 (123) |
| *oxicams (m01ac)* | 1.6 (1005) | 1.6 (975) | 2.6 (30) | 1.7 (726) | 1.5 (279) |
| *drugs used in opioid dependence (n07bc)* | 1.4 (844) | 1.4 (814) | 2.6 (30) | 1.4 (587) | 1.4 (257) |
| *cytotoxic antibiotics and related substances (l01d)* | 2.7 (1627) | 2.7 (1598) | 2.5 (29) | 2.7 (1137) | 2.7 (490) |
| *other antimigraine preparations (n02cx)* | 1.1 (696) | 1.1 (667) | 2.5 (29) | 1.2 (505) | 1 (191) |
| *testosterone-5-alpha reductase inhibitors (g04cb)* | 1.8 (1088) | 1.8 (1060) | 2.4 (28) | 1.8 (778) | 1.7 (310) |
| *non-selective monoamine reuptake inhibitors (n06aa)* | 1.5 (893) | 1.4 (865) | 2.4 (28) | 1.4 (611) | 1.5 (282) |
| *therapeutic radiopharmaceuticals (v10)* | 1.7 (1010) | 1.6 (982) | 2.4 (28) | 1.6 (700) | 1.7 (310) |
| *benzodiazepine derivatives (n03ae)* | 1.9 (1173) | 1.9 (1146) | 2.3 (27) | 1.9 (820) | 1.9 (353) |
| *antiadrenergic agents, centrally acting (c02a)* | 1.2 (705) | 1.1 (679) | 2.2 (26) | 1.2 (511) | 1.1 (194) |
| *carbapenems (j01dh)* | 1 (584) | 0.9 (558) | 2.2 (26) | 1 (430) | 0.8 (154) |
| *coxibs (m01ah)* | 2.5 (1498) | 2.5 (1473) | 2.1 (25) | 2.5 (1064) | 2.4 (434) |
| *pituitary and hypothalamic hormones (h01)* | 1 (609) | 1 (585) | 2 (24) | 1 (425) | 1 (184) |
| *imidazole derivatives (j02ab)* | 1 (595) | 1 (571) | 2 (24) | 1 (424) | 0.9 (171) |
| *digestives, incl. enzymes (a09a)* | 1.2 (704) | 1.1 (681) | 2 (23) | 1.2 (492) | 1.2 (212) |
| *cardiac glycosides (c01a)* | 0.6 (377) | 0.6 (354) | 2 (23) | 0.6 (260) | 0.6 (117) |
| *low-ceiling diuretics, excl. thiazides (c03b)* | 0.8 (519) | 0.8 (496) | 2 (23) | 0.9 (379) | 0.8 (140) |
| *centrally acting sympathomimetics (n06ba)* | 1 (596) | 1 (573) | 2 (23) | 1 (409) | 1 (187) |
| *dipeptidyl peptidase 4 (dpp-4) inhibitors (a10bh)* | 1.3 (779) | 1.3 (757) | 1.9 (22) | 1.3 (537) | 1.3 (242) |
| *dopaminergic agents (n04b)* | 0.9 (572) | 0.9 (553) | 1.6 (19) |  |  |
| *drugs for functional gastrointestinal disorders (a03a)* | 1.2 (741) | 1.2 (723) | 1.5 (18) |  |  |
| *peripheral vasodilators (c04a)* | 1.2 (758) | 1.2 (741) | 1.5 (17) |  |  |
| *fibrates (c10ab)* | 0.9 (563) | 0.9 (546) | 1.5 (17) |  |  |
| *other antianemic preparations (b03x)* | 0.3 (190) | 0.3 (174) | 1.4 (16) |  |  |
| *antimalarials (p01b)* | 0.6 (339) | 0.5 (323) | 1.4 (16) |  |  |
| *other lipid modifying agents (c10ax)* | 1.1 (656) | 1.1 (641) | 1.3 (15) |  |  |
| *azaspirodecanedione derivatives (n05be)* | 0.8 (477) | 0.8 (462) | 1.3 (15) |  |  |
| *other alimentary tract and metabolism products (a16a)* | 0.5 (328) | 0.5 (314) | 1.2 (14) |  |  |
| *antiadrenergic agents, peripherally acting (c02c)* | 0.7 (430) | 0.7 (417) | 1.1 (13) |  |  |
| *bile acid sequestrants (c10ac)* | 0.3 (209) | 0.3 (196) | 1.1 (13) |  |  |
| *nicotinic acid and derivatives (c10ad)* | 0.8 (519) | 0.8 (506) | 1.1 (13) |  |  |
| *antiadrenal preparations (h02c)* | 0.7 (400) | 0.6 (387) | 1.1 (13) |  |  |
| *glucagon-like peptide-1 (glp-1) analogues (a10bj)* | 0.8 (481) | 0.8 (469) | 1 (12) |  |  |
| *sodium-glucose co-transporter 2 (sglt2) inhibitors (a10bk)* | 1 (629) | 1 (617) | 1 (12) |  |  |
| *barbiturates and derivatives (n03aa)* | 0.3 (191) | 0.3 (179) | 1 (12) |  |  |
| *other gout preparations (m04ac)* | 0.5 (303) | 0.5 (292) | 0.9 (11) |  |  |
| *anticholinergic agents (n04a)* | 0.8 (477) | 0.8 (466) | 0.9 (11) |  |  |
| *other anti-dementia drugs (n06dx)* | 0.7 (440) | 0.7 (429) | 0.9 (11) |  |  |
| *other anti inflammatory and anti rheumatic agents, non steroids (m01ax)* | 0.9 (522) | 0.9 (512) | 0.9 (10) |  |  |
| *selective serotonin (5ht1) agonists (n02cc)* | 0.6 (378) | 0.6 (369) | 0.8 (9) |  |  |
| *anti-parathyroid agents (h05b)* | 0.3 (153) | 0.2 (145) | 0.7 (8) |  |  |
| *hydantoin derivatives (n03ab)* | 0.3 (169) | 0.3 (161) | 0.7 (8) |  |  |
| *carboxamide derivatives (n03af)* | 0.3 (191) | 0.3 (183) | 0.7 (8) |  |  |
| *antifibrinolytics (b02a)* | 1.2 (708) | 1.2 (701) | 0.6 (7) |  |  |
| *monobactams (j01df)* | 0.2 (109) | 0.2 (102) | 0.6 (7) |  |  |
| *poly (adp-ribose) polymerase (parp) inhibitors (l01xk)* | 0.4 (250) | 0.4 (243) | 0.6 (7) |  |  |
| *other drugs for disorders of the musculo-skeletal system (m09a)* | 0.5 (308) | 0.5 (301) | 0.6 (7) |  |  |
| *other antipsychotics (n05ax)* | 0.5 (313) | 0.5 (306) | 0.6 (7) |  |  |
| *diagnostic radiopharmaceuticals (v09)* | 1.2 (725) | 1.2 (718) | 0.6 (7) |  |  |
| *thiazolidinediones (a10bg)* | 0.5 (277) | 0.5 (271) | 0.5 (6) |  |  |
| *anticholinesterases (n06da)* | 0.3 (193) | 0.3 (187) | 0.5 (6) |  |  |
| *fatty acid derivatives (n03ag)* | 0.4 (236) | 0.4 (231) | 0.4 (5) |  |  |
| *bile therapy (a05a)* | 0.2 (152) | 0.2 (148) | 0.3 (4) |  |  |
| *antiobesity preparations, excl. diet products (a08a)* | 0.1 (59) | 0.1 (55) | 0.3 (4) |  |  |
| *direct thrombin inhibitors (b01ae)* | 0.2 (146) | 0.2 (142) | 0.3 (4) |  |  |
| *other hematological agents (b06a)* | 0.2 (127) | 0.2 (123) | 0.3 (4) |  |  |
| *other antimycotics for systemic use (j02ax)* | 0.2 (131) | 0.2 (127) | 0.3 (4) |  |  |
| *immunoglobulins (j06b)* | 0.1 (43) | 0.1 (39) | 0.3 (4) |  |  |
| *retinoids for cancer treatment (l01xf)* | 0.3 (158) | 0.3 (154) | 0.3 (4) |  |  |
| *antidiarrheal microorganisms (a07f)* | 0.1 (81) | 0.1 (78) | 0.3 (3) |  |  |
| *antineoplastic cell and gene therapy (l01xl)* | 0.2 (97) | 0.2 (94) | 0.3 (3) |  |  |
| *combinations of antineoplastic agents (l01xy)* | 0.1 (59) | 0.1 (56) | 0.3 (3) |  |  |
| *increase uric acid secretion (m04ab)* | 0.1 (47) | 0.1 (44) | 0.3 (3) |  |  |
| *calcitonin gene-related peptide (cgrp) antagonists (n02cd)* | 0.1 (37) | 0.1 (34) | 0.3 (3) |  |  |
| *indole derivatives (n05ae)* | 0.1 (60) | 0.1 (57) | 0.3 (3) |  |  |
| *agents against leishmaniasis and trypanosomiasis (p01c)* | 0.1 (71) | 0.1 (68) | 0.3 (3) |  |  |
| *ectoparasiticides, including. scabicides (p03a)* | 0.4 (268) | 0.4 (265) | 0.3 (3) |  |  |
| *intestinal adsorbents (a07b)* | 0.1 (55) | 0.1 (53) | 0.2 (2) |  |  |
| *other diuretics (c03x)* | 0.1 (51) | 0.1 (49) | 0.2 (2) |  |  |
| *antifungals for systemic use (d01b)* | 0.2 (98) | 0.2 (96) | 0.2 (2) |  |  |
| *antipsoriatics for systemic use (d05b)* | 0.1 (35) | 0.1 (33) | 0.2 (2) |  |  |
| *other sex hormones and modulators of the genital system (g03x)* | 0.1 (78) | 0.1 (76) | 0.2 (2) |  |  |
| *antithyroid preparations (h03b)* | 0.2 (106) | 0.2 (104) | 0.2 (2) |  |  |
| *drugs for treatment of lepra (j04b)* | 0.1 (50) | 0.1 (48) | 0.2 (2) |  |  |
| *viral vaccines (j07b)* | 0 (30) | 0 (28) | 0.2 (2) |  |  |
| *penicillamine and similar agents (m01cc)* | 0 (14) | 0 (12) | 0.2 (2) |  |  |
| *drugs used in alcohol dependence (n07bb)* | 0.1 (71) | 0.1 (69) | 0.2 (2) |  |  |
| *prostaglandins (a02bb)* | 0.1 (52) | 0.1 (51) | 0.1 (1) |  |  |
| *other blood glucose lowering drugs, excl. insulins (a10bx)* | 0.2 (97) | 0.2 (96) | 0.1 (1) |  |  |
| *anabolic steroids (a14a)* | 0 (28) | 0 (27) | 0.1 (1) |  |  |
| *other antithrombotic agens (b01ax)* | 0.1 (62) | 0.1 (61) | 0.1 (1) |  |  |
| *pyrimidine derivatives (c02dc)* | 0 (27) | 0 (26) | 0.1 (1) |  |  |
| *uterotonics (g02a)* | 0.1 (59) | 0.1 (58) | 0.1 (1) |  |  |
| *other cephalosporins and penems (j01di)* | 0 (5) | 0 (4) | 0.1 (1) |  |  |
| *antibiotics (j02aa)* | 0 (15) | 0 (14) | 0.1 (1) |  |  |
| *methylhydrazines (l01xb)* | 0 (6) | 0 (5) | 0.1 (1) |  |  |
| *proteasome inhibitors (l01xg)* | 0.1 (48) | 0.1 (47) | 0.1 (1) |  |  |
| *pyrazolones (n02bb)* | 1.2 (735) | 1.2 (734) | 0.1 (1) |  |  |
| *other analgesics and antipyretics (n02bg)* | 0 (14) | 0 (13) | 0.1 (1) |  |  |
| *barbiturates, plain (n05ca)* | 0 (10) | 0 (9) | 0.1 (1) |  |  |
| *aldehydes and derivatives (n05cc)* | 0 (6) | 0 (5) | 0.1 (1) |  |  |
| *other psychostimulants and nootropics (n06bx)* | 0 (9) | 0 (8) | 0.1 (1) |  |  |
| *antinematodal agents (p02c)* | 0.1 (68) | 0.1 (67) | 0.1 (1) |  |  |
| *liver therapy (a05b)* | 0.2 (112) | 0.2 (112) | 0 (0) |  |  |
| *other antidiarrheals (a07x)* | 0 (17) | 0 (17) | 0 (0) |  |  |
| *alpha glucosidase inhibitors (a10bf)* | 0 (16) | 0 (16) | 0 (0) |  |  |
| *other drugs used in diabetes (a10x)* | 0 (0) | 0 (0) | 0 (0) |  |  |
| *blood and related products (b05a)* | 0.1 (65) | 0.1 (65) | 0 (0) |  |  |
| *antiadrenergic agents, ganglion-blocking (c02b)* | 0 (0) | 0 (0) | 0 (0) |  |  |
| *thiazide derivatives (c02da)* | 0 (0) | 0 (0) | 0 (0) |  |  |
| *nitroferricyanide derivatives (c02dd)* | 0 (17) | 0 (17) | 0 (0) |  |  |
| *tyrosine hydroxylase inhibitors (c02kb)* | 0 (1) | 0 (1) | 0 (0) |  |  |
| *antihypertensives for pulmonary arterial hypertension (c02kx)* | 0 (9) | 0 (9) | 0 (0) |  |  |
| *capillary stabilizing agents (c05c)* | 0 (16) | 0 (16) | 0 (0) |  |  |
| *anti-acne preparations for systemic use (d10b)* | 0 (5) | 0 (5) | 0 (0) |  |  |
| *gonadotropins and other ovulation stimulants (g03g)* | 0 (8) | 0 (8) | 0 (0) |  |  |
| *antiandrogens (g03h)* | 0 (0) | 0 (0) | 0 (0) |  |  |
| *parathyroid hormones and analogues (h05a)* | 0 (23) | 0 (23) | 0 (0) |  |  |
| *amphenicols (j01b)* | 0 (7) | 0 (7) | 0 (0) |  |  |
| *aminosalicylic acid and derivatives (j04aa)* | 0 (8) | 0 (8) | 0 (0) |  |  |
| *antibiotics (j04ab)* | 0.1 (61) | 0.1 (61) | 0 (0) |  |  |
| *hydrazides (j04ac)* | 0 (30) | 0.1 (30) | 0 (0) |  |  |
| *thiocarbamide derivatives (j04ad)* | 0 (0) | 0 (0) | 0 (0) |  |  |
| *other drugs for treatment of tuberculosis (j04ak)* | 0 (21) | 0 (21) | 0 (0) |  |  |
| *sensitizers used in photodynamic/radiation therapy (l01xd)* | 0 (11) | 0 (11) | 0 (0) |  |  |
| *histone deacetylase (hdac) inhibitors (l01xh)* | 0 (26) | 0 (26) | 0 (0) |  |  |
| *hedgehog pathway inhibitors (l01xj)* | 0.1 (37) | 0.1 (37) | 0 (0) |  |  |
| *butylpyrozolidines (m01aa)* | 0 (0) | 0 (0) | 0 (0) |  |  |
| *fenamates (m01ag)* | 0 (4) | 0 (4) | 0 (0) |  |  |
| *gold preparations (m01cb)* | 0 (26) | 0 (26) | 0 (0) |  |  |
| *muscle relaxants, directly acting agents (m03c)* | 0 (3) | 0 (3) | 0 (0) |  |  |
| *barbituatues (n01af)* | 0 (7) | 0 (7) | 0 (0) |  |  |
| *ergot alkaloids (n02ca)* | 0 (7) | 0 (7) | 0 (0) |  |  |
| *oxazolidine derivatives (n03ac)* | 0 (0) | 0 (0) | 0 (0) |  |  |
| *succinimide derivatives (n03ad)* | 0 (0) | 0 (0) | 0 (0) |  |  |
| *other anti-parkinson's drugs (n04c)* | 0 (0) | 0 (0) | 0 (0) |  |  |
| *thioxanthene derivatives (n05af)* | 0 (1) | 0 (1) | 0 (0) |  |  |
| *diphenylbutylpiperidine derivatives (n05ag)* | 0 (1) | 0 (1) | 0 (0) |  |  |
| *benzamides (n05al)* | 0.1 (88) | 0.1 (88) | 0 (0) |  |  |
| *lithium (n05an)* | 0 (9) | 0 (9) | 0 (0) |  |  |
| *carbamates (n05bc)* | 0 (4) | 0 (4) | 0 (0) |  |  |
| *other anxiolitics (n05bx)* | 0 (0) | 0 (0) | 0 (0) |  |  |
| *piperidinedione derivatives (n05ce)* | 0 (0) | 0 (0) | 0 (0) |  |  |
| *orexin receptor antagonists (n05cj)* | 0 (18) | 0 (18) | 0 (0) |  |  |
| *monoamine oxidase inhibitors, non-selective (n06af)* | 0 (2) | 0 (2) | 0 (0) |  |  |
| *monoamine oxidase a inhibitors (n06ag)* | 0 (0) | 0 (0) | 0 (0) |  |  |
| *antivertigo preparations (n07c)* | 0.1 (32) | 0.1 (32) | 0 (0) |  |  |
| *other nervous system drugs (n07xx)* | 0 (10) | 0 (10) | 0 (0) |  |  |
| *antitrematodals (p02b)* | 0 (1) | 0 (1) | 0 (0) |  |  |
| *lung surfactants (r07aa)* | 0 (0) | 0 (0) | 0 (0) |  |  |
| *respiratory stimulus (r07ab)* | 0 (1) | 0 (1) | 0 (0) |  |  |
| *other respiratory system products (r07ax)* | 0 (2) | 0 (2) | 0 (0) |  |  |
| *contrast media (v08)* | 0 (2) | 0 (2) | 0 (0) |  |  |

**Supplemental Table 7: Highest Achieved Average G-means Values with Repeated Cross-Validation for Elastic Net, Gradient-Boosted Trees, and Random Forest Models Using Different Sampling Approaches**

From left to right, the first column represents an implemented sampling or weighting approach, followed by their resulting average G-means values (standard deviation) from the best performing hyper-parameter combination with repeated cross-validation specific to each Machine Learning Model implemented. Rows with numeric designations of ratios (30:1, 20:1, and 10:1), representing weighting ratios attempted for the positive class.

| **Sampling** | **Elastic Net** | **Gradient Boosted Trees** | **Random Forest** |
| --- | --- | --- | --- |
| *Down* | **0.631** | **0.661 (0.027)** | **0.694 (0.028)** |
| *Up* | 0.625 | 0.612 (0.027) | 0.003 (0.003) |
| *Smote* | 0.629 | 0.0153 (0.007) | 0.007 (0.004) |
| *Rose* | 0.613 | 0.055 (0.020) | 0.144 (0.028) |
| *No Resampling* | 0.008 | 0.0153 (0.007) | 0.003 (0.003) |
| *30:1* | 0.410 | 0.368 (0.026) | 0.007 (0.004) |
| *20:1* | 0.296 | 0.242 (0.028) | 0.006 (0.004) |
| *10:1* | 0.008 | 0.124 (0.023) | 0.007 (0.004) |

**Supplemental Table 8: Feature Importance Measures Utilized to for Feature Selection**

From the left to right, the first column represents a potential feature, followed by feature importance metrics for elastic net regularization, gradient boosted trees, and random forest models. These feature importance metrics were for models including all potential features; the total designated below each model name is the number of features having importance greater than 0.

| **Feature** | **Elastic Net**  *n= 43* | **xgbTree**  *n= 62* | **Random  Forest**  *n= 186* |
| --- | --- | --- | --- |
| *weight loss* | 100 | 100 | 100 |
| *fluid/electrolyte disorder* | 49.2684069 | 21.5402498 | 97.5252437 |
| *metastatic cancer* | 62.5534412 | 38.7831323 | 36.9807742 |
| *cardiac arrythmia* | 30.952397 | 33.8500092 | 41.0306796 |
| *pulmonary circulation disorders* | 59.989521 | 7.9694486 | 26.3077915 |
| *lung cancer* | 51.496126 | 15.18685 | 22.2878889 |
| *other cough suppressants (r05db)* | 48.5857762 | 0 | 26.1513478 |
| *high nci index tier* | 9.6318388 | 3.6214865 | 53.7637025 |
| *expectorants (r05ca)* | 53.8528449 | 0 | 12.8513873 |
| *opioids (n02a)* | 21.8418076 | 6.8103635 | 35.5565674 |
| *macrolides, lincosamides and streptogramins (j01f)* | 46.3790104 | 3.8055743 | 12.3430204 |
| *combination therapy* | 29.2694212 | 0 | 29.950067 |
| *platelet aggregation agents (b01ac)* | 21.3581758 | 6.8415211 | 22.6167576 |
| *beta blocking agents (c07)* | 13.8906121 | 8.6526103 | 26.8589168 |
| *neurological disorders* | 10.2744357 | 4.6232474 | 31.3729456 |
| *paralysis* | 40.4602196 | 0 | 4.4821342 |
| *benzodiazepine derivatives (n05ba)* | 2.3040965 | 6.69861 | 34.1929758 |
| *chronic pulmonary disease* | 13.9972248 | 8.7515203 | 15.3200017 |
| *halogenated hydrocarbons (n01ab)* | 30.3366231 | 0 | 7.3283212 |
| *triazole and tetrazole derivatives (j02ac)* | 27.480352 | 0 | 6.6781145 |
| *antihistamines for systemic use (r06a)* | 14.3159519 | 5.7824102 | 13.0710577 |
| *proton pump inhibitors (a02bc)* | 7.0263442 | 3.0243658 | 22.9158312 |
| *acute myocardial infarction* | 25.2112121 | 0 | 7.6620901 |
| *antimetabolites (l01b)* | 15.8111014 | 2.2895098 | 13.7603526 |
| *anilides (n02be)* | 7.3194304 | 1.0396333 | 23.041478 |
| *coagulopathy* | 5.8014686 | 11.3536732 | 12.2651161 |
| *depression* | 0 | 0 | 28.3180806 |
| *other respiratory cancer* | 21.5266687 | 0 | 6.6616773 |
| *tetracyclines (j01a)* | 22.4348125 | 0 | 4.5196776 |
| *pd-l1 monothery* | 10.2710638 | 4.1672242 | 10.891915 |
| *opium alkaloids and derivatives (r05da)* | 0 | 3.9723868 | 21.0041106 |
| *salicylic acid and derivatives (n02ba)* | 0 | 4.82921 | 20.0881024 |
| *vitamin b12 and folic acid (b03b)* | 12.2262675 | 1.4025574 | 10.8920351 |
| *antacids (a02a)* | 10.2893962 | 6.3166327 | 7.1709582 |
| *low-ceiling diuretics, excl. thiazides (c03b)* | 20.2495425 | 0 | 3.4988537 |
| *high-ceiling diuretics (c03c)* | 9.5922269 | 0 | 13.2660879 |
| *drug abuse* | 8.7987231 | 0 | 12.9194209 |
| *valvular disease* | 0 | 0 | 20.8032887 |
| *calcium channel blockers (c08)* | 0 | 4.9245701 | 13.3659122 |
| *drugs for constipation (a06aa)* | 0 | 2.2631504 | 15.5155604 |
| *benzodiazepine related drugs (n05cf)* | 9.2423774 | 0 | 8.3563674 |
| *direct factor xa inhibitors (b01af)* | 0.3212019 | 0 | 15.2073261 |
| *vitamin a and d, incl. combinations of the two (a11c)* | 6.767662 | 1.2058332 | 6.6113483 |
| *peptic ulcer disease* | 0 | 0 | 14.5740738 |
| *age 45 to 65* | 0 | 5.164416 | 8.4572311 |
| *black* | 0 | 0 | 13.392535 |
| *drugs for constipation (a06a)* | 0 | 0.7823911 | 12.5587567 |
| *rhematoid arthrisis/ collagated vascular disease* | 8.4532271 | 0 | 4.7762543 |
| *renal failue* | 0 | 0.4691916 | 12.6537271 |
| *male sex* | 0 | 2.7280482 | 10.3009971 |
| *hypothyroidism* | 0 | 0 | 12.9736095 |
| *antibiotics (r02ab)* | 8.8851048 | 0 | 4.0849186 |
| *phenothiazines (n05a_c)* | 0 | 3.1942901 | 9.3860717 |
| *heparin group (b01ab)* | 0 | 1.8082306 | 10.7045173 |
| *hmg coa reductase inhibitors (c10aa)* | 0 | 0 | 11.9602425 |
| *vitamin k and other hemostatics (b02b)* | 0 | 3.8652616 | 7.7997413 |
| *urniary frequency and incontinence (g04bd)* | 0 | 0 | 11.6508558 |
| *other antidepressants (n06ax)* | 0 | 0 | 11.4535989 |
| *potassium (a12b)* | 0 | 1.4904107 | 9.790734 |
| *age 75+* | 0 | 1.001323 | 10.2641479 |
| *age 65 to 75* | 0 | 0.9258806 | 9.7022075 |
| *uncomplicated diabetes* | 0 | 0 | 10.5105531 |
| *hispanic* | 0 | 0 | 10.4731813 |
| *complicated hypertension* | 0 | 0 | 10.4156904 |
| *drugs used in nicotine dependence (n07ba)* | 2.740445 | 0 | 7.5918136 |
| *thyroid preparations (h03a)* | 0 | 0 | 10.2426707 |
| *pd-1 monotherapy* | 0 | 2.5349812 | 7.6827813 |
| *intestinal antiinfectives (a07a)* | 0 | 5.4844952 | 4.7225995 |
| *alkylating agents (l01a)* | 0 | 0 | 10.0999179 |
| *skin cancer* | 0 | 0 | 9.9487733 |
| *corticosteroids, dermatological preparations (d07)* | 0 | 3.7581531 | 5.8920845 |
| *vitamin k antagonist (b01aa)* | 0 | 0 | 9.589791 |
| *alpha adrenoreceptor antagonists (g04ca)* | 0 | 0 | 9.5622454 |
| *renal cancer* | 0 | 0 | 9.4318256 |
| *propionic acid derviatives (m01ae)* | 0 | 0 | 9.2874517 |
| *diazepines, oxazepines, thiazepines and oxepines (n05ah)* | 0 | 0 | 9.2510485 |
| *congestive heart failure* | 2.4459891 | 0 | 6.7322846 |
| *benzodiazepine derivatives (n05cd)* | 0 | 0.3887676 | 8.7167616 |
| *cardiac stimulants excl. cardiac glycosides (c01c)* | 0 | 0.9666127 | 8.0968856 |
| *other antiemetics (a04ad)* | 0 | 0 | 9.0394832 |
| *immunostimulants (l03a)* | 2.6308159 | 0 | 6.3902365 |
| *medium nci index tier* | 0 | 1.0278306 | 7.9017568 |
| *h2ras (a02ba)* | 0 | 0.4062847 | 8.5055404 |
| *muscle relaxants, peripherally acting agents (m03a)* | 0 | 0.204848 | 8.6001882 |
| *parasympathomimetics (n07a)* | 0 | 0 | 8.7426521 |
| *platinum compounds (l01xa)* | 0 | 1.0208382 | 7.6437333 |
| *antithrombotic enzymes (b01ad)* | 0 | 0 | 8.6469763 |
| *obesity* | 0 | 1.8569071 | 6.7828719 |
| *other urologicals (g04bx)* | 0 | 0 | 8.5263544 |
| *serotonin (5ht3) antagonists (a04aa)* | 0 | 0.5906541 | 7.9090309 |
| *plant alkaloids and other natural products (l01c)* | 0 | 0 | 8.4199152 |
| *uncomplicated hypertension* | 0 | 0 | 8.3549866 |
| *calcium (a12a)* | 0 | 2.5627267 | 5.3028953 |
| *opioid anesthetics (n01ah)* | 0 | 0.6782786 | 7.1489445 |
| *protein kinase inhibitors (l01e)* | 4.0479547 | 0 | 3.7492897 |
| *other drugs affecting bone structure and mineralization (m05bx)* | 0 | 0 | 7.6886448 |
| *liver disease* | 0 | 0.5441457 | 7.1121585 |
| *asian* | 0 | 0 | 7.6522389 |
| *melatonin receptor agonists (n05ch)* | 0 | 0 | 7.5142203 |
| *oral cancer* | 0 | 0 | 7.5065164 |
| *white* | 0 | 0 | 7.4655747 |
| *low-ceiling diuretics, thiazides (c03a)* | 0 | 0 | 7.3124755 |
| *beta-lactam antibacterials, penicillins (j01c)* | 0 | 0.6911532 | 6.6009652 |
| *antiarrhythmics, class i and iii (c01b)* | 0 | 0.7298128 | 6.4568801 |
| *muscle relaxants, centrally acting agents (m03b)* | 0 | 0.5949953 | 6.4816952 |
| *agents acting on the renin-angiotensin system (c09)* | 0 | 0 | 7.0427811 |
| *cephalosporins 1_4 (j01db_e)* | 0 | 1.4572802 | 5.5514891 |
| *other cardiac preparations (c01e)* | 0 | 0 | 7.0063555 |
| *antiinfectives and antiseptics, excl. combinations with corticosteroids (g01a)* | 0 | 1.5202755 | 5.4115841 |
| *alcohol abuse* | 0 | 0 | 6.8418331 |
| *peripheral vascular diseases* | 0 | 0 | 6.7242063 |
| *other digestive cancer* | 0 | 0 | 6.699682 |
| *bisphosphonates (m05ba)* | 0 | 0 | 6.6295912 |
| *aldosterone antagonists and other potassium-sparing agents (c03d)* | 0 | 0 | 6.5548413 |
| *belladonna and derivatives, plain (a03b)* | 0 | 0 | 6.5002302 |
| *immunosuppressants (l04a)* | 0 | 0 | 6.4770991 |
| *corticosteroids for systemic use, plain (h02a)* | 0 | 1.2208198 | 5.1408815 |
| *insulins and analogues (a10a)* | 0 | 0 | 6.2530338 |
| *diphenylmethane derivatives (n05bb)* | 0 | 0 | 6.2394905 |
| *blood loss anemia* | 0 | 0 | 6.2222012 |
| *breast cancer* | 0 | 0 | 6.1541393 |
| *acetic acid derivatives (m01ab)* | 0 | 0 | 6.1287714 |
| *antiseptics (r02aa)* | 0 | 1.0237726 | 5.1011329 |
| *hydrazinophthalazine derivatives (c02db)* | 0 | 0 | 6.0914305 |
| *other hypnotics and sedatives (n05cm)* | 0 | 0 | 6.0600008 |
| *other general anesthetics (n01ax)* | 0 | 0.2502081 | 5.5699227 |
| *biguanides (a10ba)* | 0 | 0 | 5.7301385 |
| *liver cancer* | 0 | 0 | 5.6326535 |
| *mesothelial soft tissue cancer* | 0 | 0 | 5.4969167 |
| *uric acid production prevention (m04aa)* | 0 | 0 | 5.4573229 |
| *sulfonamides and trimethoprim (j01e)* | 0 | 0 | 5.4499482 |
| *other gynecologicals (g02c)* | 0 | 0 | 5.4474573 |
| *intestinal antiinflammatory agents (a07e)* | 0 | 0.426667 | 4.9575485 |
| *central nervous system cancer* | 0 | 0 | 5.3749872 |
| *blood cancer* | 0 | 0 | 5.3006974 |
| *selective serotonin reuptake inhibitors (n06ab)* | 0 | 0 | 5.1421897 |
| *other antimigraine preparations (n02cx)* | 0 | 0 | 5.0789056 |
| *other antiepileptics (n03ax)* | 0 | 0.7948772 | 4.2772695 |
| *propulsives (a03f)* | 0 | 0 | 5.0420064 |
| *bladder cancer* | 0 | 0 | 5.0000853 |
| *other antineoplastic agents (l01xx)* | 0 | 0 | 4.7861269 |
| *direct acting antivirals (j05a)* | 0 | 0 | 4.5636117 |
| *acidifiers (g04ba)* | 0 | 0 | 4.5330331 |
| *glycogenolytic hormones (h04a)* | 0 | 0 | 4.4193294 |
| *gabapentinoids (n02bf)* | 0 | 0 | 4.3861874 |
| *complicatd diabetes* | 0 | 0 | 4.3401135 |
| *therapeutic radiopharmaceuticals (v10)* | 0 | 0 | 4.2849558 |
| *antipropulsives (a07d)* | 0 | 0 | 4.2568184 |
| *iron preparations (b03a)* | 0 | 0 | 4.2058308 |
| *history of myocardial infarction* | 0 | 0 | 4.1640242 |
| *deficiency anemia* | 0 | 0 | 4.1590042 |
| *testosterone-5-alpha reductase inhibitors (g04cb)* | 0 | 0 | 3.8697775 |
| *colorectal cancer* | 0 | 0 | 3.7482164 |
| *mucolytics (r05cb)* | 0 | 0 | 3.686508 |
| *ascorbic acid (vitamin c), incl. combinations (a11g)* | 0 | 0 | 3.67256 |
| *cancer endocrine therapies (l02)* | 0 | 0 | 3.5543221 |
| *vitamin b1, plain and in combination with vitamin b6 and b12 (a11d)* | 0 | 0 | 3.4876644 |
| *vitamin b1, plain and in combination with vitamin b6 and b13 (a11d)* | 0 | 0 | 3.4876644 |
| *vitamin b1, plain and in combination with vitamin b6 and b14 (a11d)* | 0 | 0 | 3.4876644 |
| *butyrophenone derivatives (n05ad)* | 0 | 0 | 3.4043477 |
| *other drugs for peptic ulcer and gastro-oesophageal reflux disease (gord) (a02bx)* | 0 | 0 | 3.1084538 |
| *aminoglycoside antibacterials (j01g)* | 0 | 0 | 2.9879679 |
| *cytotoxic antibiotics and related substances (l01d)* | 0 | 0 | 2.9731954 |
| *other antibacterials (j01x)* | 0 | 0 | 2.7182918 |
| *ctla4 monotherapy* | 0 | 0 | 2.5386185 |
| *imidazole derivatives (j02ab)* | 0 | 0 | 2.4980144 |
| *agents against amoebiasis and other protozoan diseases (p01a)* | 0 | 0 | 2.4693046 |
| *vasodilators used in cardiac diseases (c01d)* | 0 | 0 | 2.3176423 |
| *coxibs (m01ah)* | 0 | 0 | 2.2391928 |
| *antiadrenergic agents, centrally acting (c02a)* | 0 | 0 | 2.2231204 |
| *sulfonylureas (a10bb)* | 0 | 0 | 2.0942277 |
| *nasal decongestants for systemic use (r01b)* | 0 | 0.1442034 | 1.9329794 |
| *sex hormones (g03a_g)* | 0 | 0 | 1.9819525 |
| *cardiac glycosides (c01a)* | 0 | 0 | 1.9055197 |
| *drugs used in opioid dependence (n07bc)* | 0 | 0 | 1.8634575 |
| *benzodiazepine derivatives (n03ae)* | 0 | 0 | 1.7972903 |
| *erectile dysfunction drugs (g04be)* | 0 | 0 | 1.6735358 |
| *carbapenems (j01dh)* | 0 | 0 | 1.5798106 |
| *non-selective monoamine reuptake inhibitors (n06aa)* | 0 | 0 | 1.5047977 |
| *oxicams (m01ac)* | 0 | 0 | 1.2831591 |
| *xanthine derivatives (n06bc)* | 0 | 0 | 1.2334475 |
| *dipeptidyl peptidase 4 (dpp-4) inhibitors (a10bh)* | 0 | 0 | 0.9829691 |
| *digestives, incl. enzymes (a09a)* | 0 | 0 | 0.8666009 |
| *centrally acting sympathomimetics (n06ba)* | 0 | 0 | 0.7234967 |
| *psychosis* | 0 | 0 | 0.6154231 |
| *aids* | 0 | 0 | 0.4990585 |
| *pituitary and hypothalamic hormones (h01)* | 0 | 0 | 0.2865902 |
| *bacterial vaccines (j07a)* | 0 | 0 | 0 |
|  |  |  |  |

**Supplemental Table 9: Model Performance Measures for Selected Initial Models Using Training and Testing Data**

From left to right, the first column lists the machine learning model used, followed the selected hyper-parameters for each model, an indication of training or testing dataset for subsequent reported results, and different performance metrics in each subsequent column.

| **Model** | **Tuning** | **Data Set** | **AUC** | **PRAUC** | **Sensitivity** | **Specificity** | **G-means** | **Accuracy** | **Balanced  Accuracy** | **F1** | **Positive  Predictive Value** | **Negative  Predictive Value** |
| --- | --- | --- | --- | --- | --- | --- | --- | --- | --- | --- | --- | --- |
| **Elastic Net** | Alpha= 0.6 | Train Data | 0.741 | 0.065 | 0.641 | 0.726 | 0.682 | 0.725 | 0.684 | 0.081 | 0.043 | 0.991 |
|  | Lambda= 0.0359 | Test Data | 0.717 | 0.057 | 0.599 | 0.723 | 0.658 | 0.721 | 0.661 | 0.078 | 0.042 | 0.989 |
| **Gradient Boosted Trees** | Max depth= 5 Eta= 0.05 | Train Data | 0.753 | 0.074 | 0.710 | 0.666 | 0.688 | 0.667 | 0.688 | 0.075 | 0.039 | 0.992 |
|  | Min Child Wt= 40 | Test Data | 0.721 | 0.058 | 0.687 | 0.659 | 0.673 | 0.660 | 0.673 | 0.074 | 0.039 | 0.990 |
| **Random Forest** | MTRY= 40 | Train Data | 0.855 | 0.188 | 0.875 | 0.638 | 0.747 | 0.643 | 0.757 | 0.085 | 0.045 | 0.996 |
|  | Min Node Size= 50 | Test Data | 0.721 | 0.058 | 0.714 | 0.633 | 0.672 | 0.634 | 0.673 | 0.072 | 0.038 | 0.991 |

**Supplemental Table 10: Feature Importance and Mean SHAP Values for all Features Included in Final Elastic Net Logistic Regression Model**

From left to right, the first column represents feature names, followed by their scaled importance values, and their mean SHAP values.

| **Feature** | **Importance** | **Abs SHAP** |
| --- | --- | --- |
| *low-ceiling diuretics, excl. thiazides (c03b)* | 100 | 0.003962705 |
| *other respiratory cancer* | 79.978481 | 0.006035649 |
| *halogenated hydrocarbons (n01ab)* | 70.439085 | 0.005411293 |
| *triazole and tetrazole derivatives (j02ac)* | 55.749998 | 0.007506976 |
| *antibiotics (r02ab)* | 54.393901 | 0.013085074 |
| *weight loss* | 52.980597 | 0.035937065 |
| *combination therapy* | 50.717986 | 0.015767719 |
| *paralysis* | 48.137682 | 0.005220098 |
| *lung cancer* | 47.063012 | 0.042766905 |
| *acute myocardial infarction* | 46.517628 | 0.005283357 |
| *other cough suppressants (r05db)* | 45.990021 | 0.009641795 |
| *expectorants (r05ca)* | 44.521825 | 0.012496574 |
| *pulmonary circulation disorders* | 41.865697 | 0.013063296 |
| *tetracyclines (j01a)* | 40.882878 | 0.007582927 |
| *metastatic cancer* | 40.459376 | 0.04052773 |
| *antihistamines for systemic use (r06a)* | 36.7047 | 0.042029138 |
| *macrolides, lincosamides and streptogramins (j01f)* | 33.232348 | 0.012591691 |
| *protein kinase inhibitors (l01e)* | 33.092561 | 0.011194423 |
| *benzodiazepine related drugs (n05cf)* | 32.886787 | 0.005826884 |
| *pd-l1 monotherapy* | 31.811315 | 0.020387642 |
| *immunostimulants (l03a)* | 27.349471 | 0.009072088 |
| *fluid/electrolyte disorder* | 27.113567 | 0.022135359 |
| *rhematoid arthrisis/ collagated vascular disease* | 25.833931 | 0.004507138 |
| *antimetabolites (l01b)* | 22.543875 | 0.016774652 |
| *drugs used in nicotine dependence (n07ba)* | 22.26174 | 0.004447952 |
| *opioids (n02a)* | 19.378466 | 0.020728255 |
| *vitamin a and d, incl. combinations of the two (a11c)* | 17.713816 | 0.008140229 |
| *platelet aggregation agents (b01ac)* | 14.711301 | 0.008647217 |
| *cardiac arrythmia* | 14.329703 | 0.012152398 |
| *coagulopathy* | 12.797336 | 0.005641783 |
| *congestive heart failure* | 12.130514 | 0.004014356 |
| *high-ceiling diuretics (c03c)* | 11.991724 | 0.005628369 |
| *beta blocking agents (c07)* | 11.968076 | 0.00997196 |
| *drug abuse* | 11.14889 | 0.003202192 |
| *antacids (a02a)* | 9.770996 | 0.005614517 |
| *chronic pulmonary disease* | 9.563692 | 0.008269828 |
| *anilides (n02be)* | 9.164656 | 0.01055847 |
| *benzodiazepine derivatives (n05ba)* | 8.895992 | 0.00770177 |
| *direct factor xa inhibitors (b01af)* | 6.417019 | 0.002205116 |
| *proton pump inhibitors (a02bc)* | 4.518298 | 0.003930698 |
| *neurological disorders* | 4.397542 | 0.001718551 |
| *high nci index tier* | 0 | 0 |
| *vitamin b12 and folic acid (b03b)* | 0 | 0 |

**Supplemental Table 11: Feature Importance and Mean SHAP Values for all Features Included in Final Gradient Boosted Tree Model**

From left to right, the first column represents feature names, followed by their scaled importance values, and their mean SHAP values.

| **Feature** | **Importance** | **Abs SHAP** |
| --- | --- | --- |
| *weight loss* | 100 | 0.043913658 |
| *metastatic cancer* | 38.8809216 | 0.055484392 |
| *cardiac arrythmia* | 36.6763209 | 0.031276774 |
| *fluid/electrolyte disorder* | 21.1798952 | 0.028169342 |
| *lung cancer* | 17.2419151 | 0.032535917 |
| *coagulopathy* | 11.3768465 | 0.01374921 |
| *beta blocking agents (c07)* | 9.9895226 | 0.023580889 |
| *pd-1 monotherapy* | 9.4982105 | 0.022956469 |
| *benzodiazepine derivatives (n05ba)* | 9.1657485 | 0.015808127 |
| *chronic pulmonary disease* | 8.9534575 | 0.003545185 |
| *calcium channel blockers (c08)* | 8.6445719 | 0.011020189 |
| *opioids (n02a)* | 8.2967751 | 0.019405794 |
| *salicylic acid and derivatives (n02ba)* | 7.9288999 | 0.010501812 |
| *pd-l1 monotherapy* | 7.1028158 | 0.023694406 |
| *antacids (a02a)* | 7.0280813 | 0.011196007 |
| *antihistamines for systemic use (r06a)* | 6.7571069 | 0.01936975 |
| *age 45 to 65* | 5.9966458 | 0.011412896 |
| *pulmonary circulation disorders* | 5.6348622 | 0.003794227 |
| *heparin group (b01ab)* | 5.6265813 | 0.00336583 |
| *intestinal antiinfectives (a07a)* | 5.5983457 | 0.007107449 |
| *antimetabolites (l01b)* | 5.5395596 | 0.015799303 |
| *opium alkaloids and derivatives (r05da)* | 5.0509387 | 0.013773098 |
| *corticosteroids, dermatological preparations (d07)* | 4.8801016 | 0.014721621 |
| *phenothiazines (n05a_c)* | 4.7668202 | 0.011132645 |
| *high nci index tier* | 4.7233164 | 0.006689047 |
| *neurological disorders* | 4.60634 | 0.007448098 |
| *macrolides, lincosamides and streptogramins (j01f)* | 4.4269449 | 0.008129098 |
| *male sex* | 4.0241963 | 0.004949683 |
| *other antiepileptics (n03ax)* | 3.9201487 | 0.010633931 |
| *proton pump inhibitors (a02bc)* | 3.8708671 | 0.003223345 |
| *drugs for constipation (a06aa)* | 3.622517 | 0.008122599 |
| *antiarrhythmics, class i and iii (c01b)* | 3.6133288 | 0.005581291 |
| *platinum compounds (l01xa)* | 3.5591093 | 0.011472226 |
| *potassium (a12b)* | 3.3970463 | 0.008956251 |
| *vitamin k and other hemostatics (b02b)* | 3.2045136 | 0.007979777 |
| *antiinfectives and antiseptics, excl. combinations with corticosteroids (g01a)* | 3.1400789 | 0.007964596 |
| *obesity* | 3.0536034 | 0.006179805 |
| *anilides (n02be)* | 3.0496805 | 0.003993046 |
| *age 75+* | 2.9317869 | 0.003277141 |
| *cephalosporins 1_4 (j01db_e)* | 2.8982328 | 0.008174595 |
| *serotonin (5ht3) antagonists (a04aa)* | 2.8295823 | 0.008418643 |
| *calcium (a12a)* | 2.7989794 | 0.00614966 |
| *cardiac stimulants excl. cardiac glycosides (c01c)* | 2.7185804 | 0.007425725 |
| *muscle relaxants, peripherally acting agents (m03a)* | 2.5751808 | 0.008357394 |
| *medium nci index tier* | 2.0427293 | 0.007236947 |
| *nasal decongestants for systemic use (r01b)* | 2.0424505 | 0.008211189 |
| *opioid anesthetics (n01ah)* | 1.8471577 | 0.002298396 |
| *benzodiazepine derivatives (n05cd)* | 1.7786859 | 0.004638645 |
| *vitamin a and d, incl. combinations of the two (a11c)* | 1.7044006 | 0.00453034 |
| *h2ras (a02ba)* | 1.6843158 | 0.005390338 |
| *platelet aggregation agents (b01ac)* | 1.6085386 | 0.003144239 |
| *liver disease* | 1.5809737 | 0.004027564 |
| *antiseptics (r02aa)* | 1.5674707 | 0.004701123 |
| *corticosteroids for systemic use, plain (h02a)* | 1.4781903 | 0.009019817 |
| *drugs for constipation (a06a)* | 1.3134504 | 0.001463921 |
| *intestinal antiinflammatory agents (a07e)* | 1.16741 | 0.003766144 |
| *age 65 to 75* | 1.0419664 | 0.003961847 |
| *vitamin b12 and folic acid (b03b)* | 0.8256285 | 0.001346694 |
| *muscle relaxants, centrally acting agents (m03b)* | 0.5891905 | 0.001778936 |
| *beta-lactam antibacterials, penicillins (j01c)* | 0 | 0 |
| *other general anesthetics (n01ax)* | 0 | 0 |
| *renal failure* | 0 | 0 |

**Supplemental Table 12: Feature Importance and Mean SHAP Values for all Features Included in Final Random Forest Model**

From left to right, the first column represents feature names, followed by their scaled importance values, and their mean SHAP values.

| **Feature** | **Importance** | **Abs SHAP** |
| --- | --- | --- |
| *weight loss* | 100 | 0.020398801 |
| *fluid/electrolyte disorder* | 88.995438 | 0.026483788 |
| *metastatic cancer* | 41.856034 | 0.018044858 |
| *cardiac arrythmia* | 40.673228 | 0.007861188 |
| *high nci index tier* | 40.45783 | 0.009557228 |
| *opioids (n02a)* | 33.344345 | 0.009645161 |
| *combination therapy* | 32.033632 | 0.008827189 |
| *benzodiazepine derivatives (n05ba)* | 30.372812 | 0.012849074 |
| *neurological disorders* | 29.701872 | 0.006892134 |
| *other cough suppressants (r05db)* | 29.537173 | 0.005915548 |
| *anilides (n02be)* | 26.667772 | 0.006730965 |
| *proton pump inhibitors (a02bc)* | 26.272747 | 0.006936962 |
| *platelet aggregation agents (b01ac)* | 25.573095 | 0.008264491 |
| *lung cancer* | 25.216763 | 0.00997339 |
| *depression* | 24.649583 | 0.006539573 |
| *opium alkaloids and derivatives (r05da)* | 23.311955 | 0.005572944 |
| *pulmonary circulation disorders* | 23.241148 | 0.005815862 |
| *beta blocking agents (c07)* | 22.880647 | 0.007752666 |
| *chronic pulmonary disease* | 21.859991 | 0.005866023 |
| *male sex* | 20.821118 | 0.005631222 |
| *drugs for constipation (a06aa)* | 19.474339 | 0.006892481 |
| *antihistamines for systemic use (r06a)* | 18.442597 | 0.007692041 |
| *direct factor xa inhibitors (b01af)* | 17.967689 | 0.004470047 |
| *antimetabolites (l01b)* | 17.847318 | 0.006441498 |
| *calcium channel blockers (c08)* | 17.731907 | 0.004353769 |
| *valvular disease* | 17.407316 | 0.004856512 |
| *age 75+* | 17.303576 | 0.003248411 |
| *black* | 17.286675 | 0.004624226 |
| *drugs for constipation (a06a)* | 17.222129 | 0.002577896 |
| *skin cancer* | 16.896931 | 0.002795045 |
| *expectorants (r05ca)* | 16.119833 | 0.003924647 |
| *macrolides, lincosamides and streptogramins (j01f)* | 15.317392 | 0.002884187 |
| *uncomplicated hypertension* | 15.273006 | 0.001665941 |
| *vitamin b12 and folic acid (b03b)* | 15.230075 | 0.003239166 |
| *renal failure* | 15.086956 | 0.002991627 |
| *hypothyroidism* | 14.899364 | 0.003548516 |
| *heparin group (b01ab)* | 14.83963 | 0.001893434 |
| *medium nci index tier* | 14.510127 | 0.00167077 |
| *high-ceiling diuretics (c03c)* | 14.480066 | 0.003389869 |
| *age 45 to 65* | 14.426863 | 0.002234115 |
| *pd-l1 monotherapy* | 14.418698 | 0.003812411 |
| *vitamin k and other hemostatics (b02b)* | 14.409168 | 0.003005649 |
| *salicylic acid and derivatives (n02ba)* | 14.380221 | 0.003695832 |
| *age 65 to 75* | 14.227006 | 0.004040285 |
| *benzodiazepine derivatives (n05cd)* | 13.798509 | 0.003421716 |
| *corticosteroids, dermatological preparations (d07)* | 13.644727 | 0.005683059 |
| *peptic ulcer disease* | 13.50955 | 0.002172708 |
| *platinum compounds (l01xa)* | 13.50046 | 0.001889405 |
| *phenothiazines (n05a_c)* | 13.488963 | 0.001205145 |
| *intestinal antiinflammatory agents (a07e)* | 13.429805 | 0.001474187 |
| *hmg coa reductase inhibitors (c10aa)* | 13.256121 | 0.002688825 |
| *coagulopathy* | 13.223489 | 0.00196114 |
| *white* | 13.169223 | 0.00143362 |
| *thyroid preparations (h03a)* | 13.091192 | 0.002795187 |
| *uncomplicated diabetes* | 13.069638 | 0.002705495 |
| *peripheral vascular diseases* | 13.064471 | 0.001391955 |
| *alpha adrenoreceptor antagonists (g04ca)* | 13.000693 | 0.00230019 |
| *h2ras (a02ba)* | 12.938623 | 0.002211899 |
| *vitamin a and d, incl. combinations of the two (a11c)* | 12.92029 | 0.002059601 |
| *other antidepressants (n06ax)* | 12.854182 | 0.002826557 |
| *antiarrhythmics, class i and iii (c01b)* | 12.8394313 | 1.63E-03 |
| *selective serotonin reuptake inhibitors (n06ab)* | 12.5546456 | 1.74E-03 |
| *complicated hypertension* | 12.4727387 | 1.43E-03 |
| *other antiemetics (a04ad)* | 12.2819935 | 1.89E-03 |
| *serotonin (5ht3) antagonists (a04aa)* | 12.0328345 | 1.72E-03 |
| *renal cancer* | 12.0012788 | 1.75E-03 |
| *pd-1 monotherapy* | 11.9056586 | 1.15E-03 |
| *opioid anesthetics (n01ah)* | 11.8645367 | 1.54E-03 |
| *potassium (a12b)* | 11.80159 | 1.95E-03 |
| *vitamin k antagonist (b01aa)* | 11.6506366 | 1.63E-03 |
| *benzodiazepine related drugs (n05cf)* | 11.6142718 | 2.21E-03 |
| *other digestive cancer* | 11.4361761 | 1.15E-03 |
| *antiinfectives and antiseptics, excl. combinations with corticosteroids (g01a)* | 11.4100388 | 1.57E-03 |
| *beta-lactam antibacterials, penicillins (j01c)* | 11.316274 | 1.86E-03 |
| *liver disease* | 11.1827646 | 1.35E-03 |
| *parasympathomimetics (n07a)* | 11.131987 | 8.92E-04 |
| *antithrombotic enzymes (b01ad)* | 11.0520346 | 1.15E-03 |
| *drug abuse* | 11.0472541 | 1.03E-03 |
| *diazepines, oxazepines, thiazepines and oxepines (n05ah)* | 11.0057547 | 9.38E-04 |
| *plant alkaloids and other natural products (l01c)* | 10.9206615 | 1.97E-03 |
| *hispanic* | 10.8261738 | 1.36E-03 |
| *agents acting on the renin-angiotensin system (c09)* | 10.7754165 | 1.24E-03 |
| *cardiac stimulants excl. cardiac glycosides (c01c)* | 10.6917995 | 4.04E-03 |
| *urniary frequency and incontinence (g04bd)* | 10.591822 | 2.38E-03 |
| *obesity* | 10.5820265 | 1.05E-03 |
| *corticosteroids for systemic use, plain (h02a)* | 10.5595704 | 2.67E-03 |
| *other cardiac preparations (c01e)* | 10.4426598 | 2.36E-03 |
| *cephalosporins 1_4 (j01db_e)* | 10.4102549 | 1.26E-03 |
| *acute myocardial infarction* | 10.2267108 | 1.52E-03 |
| *muscle relaxants, centrally acting agents (m03b)* | 10.1205212 | 2.03E-03 |
| *oral cancer* | 10.0783912 | 7.64E-04 |
| *gabapentinoids (n02bf)* | 10.0241613 | 1.14E-03 |
| *other antiepileptics (n03ax)* | 9.7756124 | 9.10E-04 |
| *antacids (a02a)* | 9.617047 | 1.12E-03 |
| *melatonin receptor agonists (n05ch)* | 9.5745807 | 1.05E-03 |
| *immunostimulants (l03a)* | 9.5325789 | 1.48E-03 |
| *congestive heart failure* | 9.2361935 | 1.58E-03 |
| *breast cancer* | 9.1951882 | 1.53E-03 |
| *belladonna and derivatives, plain (a03b)* | 9.1576563 | 1.23E-03 |
| *other drugs affecting bone structure and mineralization (m05bx)* | 9.0471871 | 9.98E-04 |
| *other general anesthetics (n01ax)* | 9.0287492 | 1.23E-03 |
| *deficiency anemia* | 9.0042057 | 1.04E-03 |
| *bisphosphonates (m05ba)* | 8.9774401 | 1.29E-03 |
| *low-ceiling diuretics, thiazides (c03a)* | 8.9461505 | 1.07E-03 |
| *alkylating agents (l01a)* | 8.891064 | 1.19E-03 |
| *complicatd diabetes* | 8.8424256 | 8.85E-04 |
| *antiseptics (r02aa)* | 8.6577887 | 8.52E-04 |
| *drugs used in nicotine dependence (n07ba)* | 8.5719938 | 5.76E-04 |
| *glycogenolytic hormones (h04a)* | 8.5069085 | 7.60E-04 |
| *propionic acid derviatives (m01ae)* | 8.4614272 | 2.19E-03 |
| *uric acid production prevention (m04aa)* | 8.3887179 | 6.41E-04 |
| *rhematoid arthrisis/ collagated vascular disease* | 8.385049 | 5.22E-04 |
| *other urologicals (g04bx)* | 8.37402 | 1.06E-03 |
| *hydrazinophthalazine derivatives (c02db)* | 8.3291622 | 1.46E-03 |
| *immunosuppressants (l04a)* | 8.2723267 | 1.06E-03 |
| *calcium (a12a)* | 8.1451844 | 1.13E-03 |
| *bladder cancer* | 8.1440664 | 7.40E-04 |
| *biguanides (a10ba)* | 8.1051954 | 8.41E-04 |
| *other gynecologicals (g02c)* | 8.0949577 | 1.84E-03 |
| *alcohol abuse* | 8.0399257 | 1.11E-03 |
| *protein kinase inhibitors (l01e)* | 7.9658596 | 4.63E-04 |
| *propulsives (a03f)* | 7.9354122 | 1.16E-03 |
| *muscle relaxants, peripherally acting agents (m03a)* | 7.9083525 | 1.41E-03 |
| *triazole and tetrazole derivatives (j02ac)* | 7.9059542 | 1.11E-03 |
| *intestinal antiinfectives (a07a)* | 7.8415172 | 8.47E-04 |
| *acetic acid derivatives (m01ab)* | 7.699523 | 1.63E-03 |
| *other hypnotics and sedatives (n05cm)* | 7.6608243 | 7.58E-04 |
| *insulins and analogues (a10a)* | 7.5668204 | 8.53E-04 |
| *other respiratory cancer* | 7.4917886 | 9.19E-04 |
| *direct acting antivirals (j05a)* | 7.4812871 | 5.33E-04 |
| *halogenated hydrocarbons (n01ab)* | 7.4056217 | 1.06E-03 |
| *other antibacterials (j01x)* | 7.3003826 | 5.85E-04 |
| *aldosterone antagonists and other potassium-sparing agents (c03d)* | 7.2470543 | 8.15E-04 |
| *history of myocardial infarction* | 7.0300443 | 4.98E-04 |
| *antipropulsives (a07d)* | 7.0180656 | 8.04E-04 |
| *tetracyclines (j01a)* | 6.9263021 | 4.33E-04 |
| *diphenylmethane derivatives (n05bb)* | 6.9095778 | 8.62E-04 |
| *sulfonamides and trimethoprim (j01e)* | 6.8687922 | 5.68E-04 |
| *mesothelial soft tissue cancer* | 6.8463073 | 1.16E-03 |
| *blood loss anemia* | 6.4874327 | 7.40E-04 |
| *blood cancer* | 6.2283106 | 4.41E-04 |
| *acidifiers (g04ba)* | 6.1845764 | 5.94E-04 |
| *liver cancer* | 6.094524 | 5.52E-04 |
| *ascorbic acid (vitamin c), incl. combinations (a11g)* | 5.9397606 | 3.47E-04 |
| *iron preparations (b03a)* | 5.9255107 | 9.52E-04 |
| *asian* | 5.7170806 | 9.09E-04 |
| *antibiotics (r02ab)* | 5.6935957 | 5.09E-04 |
| *colorectal cancer* | 5.5503432 | 3.64E-04 |
| *nasal decongestants for systemic use (r01b)* | 5.5353645 | 5.12E-04 |
| *other antineoplastic agents (l01xx)* | 5.4938371 | 9.33E-04 |
| *aminoglycoside antibacterials (j01g)* | 5.4221018 | 6.09E-04 |
| *paralysis* | 5.4036142 | 5.51E-04 |
| *low-ceiling diuretics, excl. thiazides (c03b)* | 5.2691233 | 2.70E-04 |
| *central nervous system cancer* | 4.9691178 | 6.39E-04 |
| *mucolytics (r05cb)* | 4.8117213 | 3.31E-04 |
| *vasodilators used in cardiac diseases (c01d)* | 4.3423049 | 4.15E-04 |
| *therapeutic radiopharmaceuticals (v10)* | 4.0856876 | 2.63E-04 |
| *cytotoxic antibiotics and related substances (l01d)* | 3.8925978 | 3.10E-04 |
| *cancer endocrine therapies (l02)* | 3.7536902 | 2.82E-04 |
| *other drugs for peptic ulcer and gastro-oesophageal reflux disease (gord) (a02bx)* | 3.645527 | 4.41E-04 |
| *other antimigraine preparations (n02cx)* | 3.5716715 | 4.54E-04 |
| *sulfonylureas (a10bb)* | 3.5522642 | 2.03E-04 |
| *butyrophenone derivatives (n05ad)* | 3.3140436 | 3.48E-04 |
| *ctla4 monotherapy* | 3.2550323 | 4.88E-04 |
| *sex hormones (g03a_g)* | 3.1871503 | 4.50E-04 |
| *benzodiazepine derivatives (n03ae)* | 3.1082093 | 1.67E-04 |
| *erectile dysfunction drugs (g04be)* | 2.9812223 | 1.43E-04 |
| *non-selective monoamine reuptake inhibitors (n06aa)* | 2.9473917 | 3.14E-04 |
| *testosterone-5-alpha reductase inhibitors (g04cb)* | 2.9444489 | 2.02E-04 |
| *agents against amoebiasis and other protozoan diseases (p01a)* | 2.6694472 | 2.15E-04 |
| *coxibs (m01ah)* | 2.6429385 | 2.06E-04 |
| *vitamin b1, plain and in combination with vitamin b6 and b12 (a11d)* | 2.5483829 | 2.16E-04 |
| *antiadrenergic agents, centrally acting (c02a)* | 2.5421065 | 2.54E-04 |
| *oxicams (m01ac)* | 2.1866443 | 1.33E-04 |
| *cardiac glycosides (c01a)* | 2.1316146 | 8.98E-05 |
| *drugs used in opioid dependence (n07bc)* | 1.9428766 | 1.34E-04 |
| *imidazole derivatives (j02ab)* | 1.8994888 | 1.70E-04 |
| *xanthine derivatives (n06bc)* | 1.8977232 | 1.50E-04 |
| *carbapenems (j01dh)* | 1.7908374 | 9.73E-05 |
| *digestives, incl. enzymes (a09a)* | 1.3661901 | 1.58E-04 |
| *dipeptidyl peptidase 4 (dpp-4) inhibitors (a10bh)* | 1.2484087 | 1.03E-04 |
| *psychosis* | 1.1324523 | 6.25E-05 |
| *aids* | 0.8821143 | 3.87E-05 |
| *pituitary and hypothalamic hormones (h01)* | 0.7957008 | 5.64E-05 |
| *centrally acting sympathomimetics (n06ba)* | 0 | 2.51E-05 |

**Supplemental Table 13: Cardiac Immune Related Adverse Event Rate Across Ranked Quantiles Used for Model Calibration Curves**

This table presents adverse event rates within each ranked quantile on testing data from each machine learning model, created for calibration curve plots. Quantiles are ordered from lowest expected average probability (Q1) to highest (Q5) for each model. From left to right, the first column is the model represented, followed by the quantile, total patients within each quantile (Total Patients), total adverse events within each quantile (Total AEs), Mean assigned probability for patients within a given quantile (Mean Predicted Probability), and the percentage of adverse events occurring within each quantile (Observed Event Rate)

| **Model** | **Quantile** | **Total  Patients (n)** | **Total  AEs (n)** | **Mean Predicted Probability** | **Observed  Event Rate (%)** |
| --- | --- | --- | --- | --- | --- |
| **Elastic Net** | Q1 | 3671 | 20 | 0.191 | 0.54 |
|  | Q2 | 3663 | 35 | 0.281 | 0.96 |
|  | Q3 | 3667 | 53 | 0.373 | 1.45 |
|  | Q4 | 3667 | 81 | 0.499 | 2.21 |
|  | Q5 | 3667 | 175 | 0.727 | 4.77 |
| **Gradient Boosted Trees** | Q1 | 3667 | 20 | 0.180 | 0.55 |
|  | Q2 | 3667 | 34 | 0.285 | 0.93 |
|  | Q3 | 3667 | 47 | 0.402 | 1.28 |
|  | Q4 | 3667 | 89 | 0.536 | 2.43 |
|  | Q5 | 3667 | 174 | 0.718 | 4.75 |
| **Random Forest** | Q1 | 3668 | 21 | 0.185 | 0.57 |
|  | Q2 | 3666 | 40 | 0.343 | 1.09 |
|  | Q3 | 3667 | 38 | 0.444 | 1.04 |
|  | Q4 | 3667 | 87 | 0.533 | 2.37 |
|  | Q5 | 3667 | 178 | 0.646 | 4.85 |

**Supplemental Table 14: Top 20 Features by Feature Importance for Each ML Model**

From left to right, the first column represents feature rank, followed by columns representing features for Elastic Net (Elastic Net Logistic Regression), Gradient Boosted Trees, and Random Forest models. Rank is based on feature importance metrics from each model, so #1 corresponds to the feature having the highest importance in each model, #2 the next highest, and so on.

*Features having asterisks (*) are in all three lists.*

*Features having (^) are in 2 of 3 lists*

|  | Elastic Net | Gradient Boosted Trees | Random Forest |
| --- | --- | --- | --- |
| *1* | *low-ceiling diuretics, excl. thiazides (c03b)* | *weight loss** | *weight loss** |
| *2* | *other respiratory cancer* | *metastatic cancer** | *fluid/electrolyte disorder ^* |
| *3* | *halogenated hydrocarbons (n01ab)* | *cardiac arrhythmia ^* | *metastatic cancer** |
| *4* | *triazole and tetrazole derivatives (j02ac)* | *fluid/electrolyte disorder ^* | *cardiac arrhythmia ^* |
| *5* | *antibiotics (r02ab)* | *lung cancer** | *high nci index tier* |
| *6* | *weight loss** | *coagulopathy* | *opioids (n02a) ^* |
| *7* | *combination therapy ^* | *beta blocking agents (c07) ^* | *combination therapy ^* |
| *8* | *paralysis* | *pd-1 monotherapy* | *benzodiazepine derivatives (n05ba) ^* |
| *9* | *lung cancer** | *benzodiazepine derivatives (n05ba) ^* | *neurological disorders* |
| *10* | *acute myocardial infarction* | *chronic pulmonary disease ^* | *other cough suppressants (r05db) ^* |
| *11* | *other cough suppressants (r05db) ^* | *calcium channel blockers (c08)* | *anilides (n02be)* |
| *12* | *expectorants (r05ca)* | *opioids (n02a) ^* | *proton pump inhibitors (a02bc)* |
| *13* | *pulmonary circulation disorders** | *salicylic acid and derivatives (n02ba)* | *platelet aggregation agents (b01ac)* |
| *14* | *tetracyclines (j01a)* | *pd-l1 monotherapy* | *lung cancer** |
| *15* | *metastatic cancer** | *antacids (a02a)* | *Depression* |
| *16* | *antihistamines for systemic use (r06a) ^* | *antihistamines for systemic use (r06a) ^* | *opium alkaloids and derivatives (r05da)* |
| *17* | *macrolides, lincosamides and streptogramins (j01f)* | *age 45 to 65* | *pulmonary circulation disorders** |
| *18* | *protein kinase inhibitors (l01e)* | *pulmonary circulation disorders** | *beta blocking agents (c07) ^* |
| *19* | *benzodiazepine related drugs (n05cf)* | *heparin group (b01ab)* | *chronic pulmonary disease ^* |
| *20* | *pd-l1 monotherapy* | *intestinal ant infectives (a07a)* | *male sex* |
