# Supplementary material for: Machine learning approaches to predict early cardiac immune-related adverse events in patients receiving immune checkpoint inhibitors
Source: Support Care Cancer. 2026 Jul 29;34(8):809. doi: 10.1007/s00520-026-10984-5 (PMC13415296; doi:10.1007/s00520-026-10984-5)
Supplement: Supplementary file 2 — (DOCX 38.8 KB) [file 520_2026_10984_MOESM2_ESM.docx]

**Supplemental Methods:**

***Section 1: TriNetx Data Requisition and Files Utilized***

Files of patient data were obtained from TriNetx.^1^ Utilizing the “Query Builder” within the TriNetx platform, all patients were queried having received Immune Checkpoint Inhibitor (ICI) therapies within the Global Collaborative Network of institutions. Specific ICI agents were queried utilizing specific RxNorm codes including: Pembrolizumab (RxNorm: 1547545), Nivolumab (RxNorm: 1597876), Dostarlimab (RxNorm:2539967), Ipililumab (RxNorm: 1094833), Atezolizumab (RxNorm: 1792776), Avelumab (RxNorm: 1875534), and Durvalumab (RxNorm: 1919503). After creating the query, we requisitioned patient data through our institution University of California Irvine which has paid access to TriNetx data.

Several different files were utilized to generate patient data for the study. The specific files, along with a summary of how they were utilized are described below. The TriNetx webpage has a detailed data dictionary, which elaborates on the structure of the data represented in each file referenced.^1^

| File Name  (as designated in TriNetx files) | Information Utilized |
| --- | --- |
| medication_ingredient.csv file | The medication ingredient file contained all documented medication exposures, with each medication represented as RxNorm codes. This file was utilized to characterize **ICI index agent**, **ICI** **index date** and **medication exposures**.   - **Index ICI agent and date** were based on the date ICI(s) first appeared in the patient’s medical record and the ICI agent associated with that exposure. Combination therapies were identified if more than 1 ICI index agent appeared at index and combination had meaningful precedent in literature. Patients with multiple ICI agents listed not intuitive for combination therapy were excluded due to lack of clarity of ICI therapy received. To ensure sufficient follow up time, patients with Index dates past December 2023 were not included in the study. - **Time on ICI Therapy** was defined as the number of days between a patients first and last ICI occurence in their medical record. This measure was utilized to validate adverse event occurrence, as cardiac inflammatory adverse events were only considered if the diagnosis occurred no later than 30 days after a patients last ICI exposure. Events not meeting this criteria were labeled as “ambiguous cardiac inflammation diagnoses.” Furthermore, to ensure the study population considered were patients receiving ICI for the study timeframe, non- adverse event patients were excluded if they did not have at least 60 days time of ICI therapy. - **Medication exposures** were limited to agents appearing in the patients’ medical record up to 90 days prior to and including the day of ICI index. RxNorm codes within this timeframe were queried as clusters belonging to broader classes of Anatomical Therapeutic Chemical (ATC) classification coding groups, referencing broader groups of pharmacologically related medications. If a patient had an exposure to an agent within a given ATC class grouping, they were indicated as positive for an exposure to that class of medications. ATC code group information is contained within the TriNetx platform, in addition to the World Health Organization’s Collaborating Centre for Drug Statistics Methodology.^2^ |
| diagnosis.csv file | The diagnosis file was utilized contains every diagnosis each has received, as represented by ICD-9 and 10 codes. This file was used to characterize **history of neoplasm diagnosis**, concurrent **comorbid conditions**, **primary cancer type**, **metastatic cancer** status, and presence of **cardiac inflammation.**   - **History of neoplasm diagnosis** was identified based on a broad set of diagnostic codes suggested by TriNetx (both ICD-9 and 10 codes), which capture all potential specific and non-specific neoplasm diagnoses. Patients having no recorded diagnosis codes in consistent with neoplasm diagnosis were excluded. - **Comorbid conditions** were characterized based on all diagnostic codes occurring up to a year prior to ICI index date. Clusters of ICD-9 and ICD-10 codes were utilized to identify the occurrence of specific comorbidities within the elixhausar comorbidity index. ICD-9 and ICD-10 codes associated for each comorbid condition have been identified and optimized by several researchers, whose diagnostic codes we utilized for our queries specific to each comorbid condition.^3,4^ - **Primary cancer type** and **metastatic cancer status** were similarly characterized utilizing diagnostic codes occurring within 1 year of ICI index date. For primary cancer type, clusters of diagnostic codes (ICD-9 and ICD-10) were identified that were associated 22 distinct primary cancers grouped based on implicated cancer location or implicated system. The first cancer diagnosis occurring in the patient’s medical record up to a year removed from index belonging to one of the pre-determined groups was utilized to characterize primary cancer type. Patients not having a diagnostic code within the pre-defined group were labeled as “other” cancers. Patients with metastatic cancer were identified if they had any diagnosis codes associated with secondary or metastatic malignancies in the same timeframe. - **Cardiac inflammation** was identified based on a cluster of identified diagnosis codes associated with cardiac inflammation identified through literature search. Occurrence of these codes in the patients medical record up to 90 days prior to ICI index and up to 90 days after was identified. Patients having these diagnosis codes up to 90 days prior to index (including day of index) were excluded from the study as having “prior cardiac inflammation”. Potential immune related cardiac adverse events were defined as the first occurrence of the selected diagnosis codes up to 90 days after the index date |
| patient.csv file | The patient file was utilized to determine many patient characteristics utilized in the study. This included patient’s **age at index**, **biological sex**, **racial/ethnic identify**, and **mortality data**   - **Age at index** was characterized utilizing the year of birth information in the file together with the index date. Patients lacking year of birth information were excluded. - **Biological sex** was characterized utilizing sex information within the file. Patients with missing sex information were excluded; There was no gender identification distinction available within in the file to consider. - **Race/Ethnicity** was characterized based within separate “race” and “ethnicity” values in the file. First ethnicity was considered, identifying all Hispanic/Latino patients. Non-hispanic patients were subsequently identified as “White”, “Black”, or “Asian” based on designations in the file. Latino patients were characterized as such, with subsequent racial identities characterizing non-Hispanic patients as one of the previously mentioned racial identifications available. Patients not belonging to one of these groups were labeled as “other” due to limited sample size - **Mortality data**, or mortality status in the patient file designated whether patients were deceased at the time of collecting the data. This information was utilized for determination of patient survival time described below in the next section |
| encounter.csv file | The patient encounter file contains documented interactions with the health care system for each patient, including in-patient, out-patient, and pharmacy based experiences.   - **Cardiac immune-related adverse events** were validated utilizing patient encounters representing inpatient and emergency room encounters within the encounter file. Adverse events were only counted if the patient had one of these encounters documented within 1 week of the date of a patients potential cardiac immune related adverse event. Events not meeting this criteria were labeled as “ambiguous cardiac inflammation diagnoses.” - **Survival time** was characterized relative to ICI index date, utilizing the encounter file combined with mortality data from the previously described patient file. If patients were deceased, their survival time was time from index to morality date. For non-deceased patients, their survival time the time from index to the time of their last documented encounter within TriNetx files. Each encounter has “start” and “stop” dates associated with them representing a broad spectrum of potential medical occurrences (any general outpatient, inpatient, or emergency services visit, interaction with pharmacies etc). Encounters can represent very broad unrealistic timeframes within the file, so the start date of a patient’s very last encounter was utilized for survival time determination in non-deceased patents to prevent over estimation of potential survival times. |

***Section 2: Implementation of Machine Learning Models***

*Execution of Model Training*

Machine learning(ML) methods were implemented in the R computing platform, utilizing models featured in the caret package.^5-8^ The optimization parameter G-means was manually created function, taking the square root of the product of sensitivity x specificity to characterize model performance. Re-sampling specifications were made within the “trainControl” function of caret, including designating a repeated cross validation approach along with the specific sampling strategies (i.e Down, Up, Smote). Tuning grids specific to each machine learning model approach specified the full set of hyper-parameter combinations to be evaluated. During model training, every combination of hyper-parameters was evaluated across all folds and repeats. Three distinct ML modeling approaches were implemented utilizing caret with the “method” argument within the train function, with designations utilized including: glmnet- elastic net logistic regression, xgbTree- gradient boosted trees, ranger- random forest modeling. The train function executed ML models, taking designated arguments for the ML approach of choice, optimization metric (G-means), sampling approach (created with trainControl), and tuningGrid.

*Evaluation of Feature Contributions to Model Predictions*

Evaluation of key features for outcome prediction utilized feature importance metrics and SHAP values. Feature importance values are computed natively within caret’s training function and describe the relative contribution of each variable to the model’s predictions. These values are generated specific to each modeling approach; For regression-based approaches they relate to the magnitude of regression coefficients and for Tree-based models they capture model performance changes with and without that feature (permutation or split-gain). To enable comparison between models, importance scores are scaled such that the most influential predictor receives a score of 100. All other features are assigned values relative to this maximum, decreasing in proportion to their contribution to predictive performance. SHAP values were generated utilizing separate R-packages including “fastshap” and “nestedcv.^7,9^” Trained ML models and the training data set were utilized to generate SHAP values with the “explain” function, performing 50 Monte Carlo simulations. Positive SHAP values indicate that a given feature contributed to a higher predicted probability of the adverse event, while negative SHAP values indicate that the feature contributed to a lower predicted probability. The absolute value of generated SHAP values described the magnitude of features contributions to predictions, with higher values meaning the feature had more influence on predictions. Given all variables were encoded as binary features, features were characterized as “positive contributors” when having that characteristic the lead to increased (more positive, increased AE risk) SHAP values.

*Predictions on Training and Testing Data*

Predictions on testing and training data utilizing trained ML models was performed utilizing the “predict” function within the caret package. Model performance was assessed based on the strength of the assigned probabilities from each model relative to the true outcome for each patient, with assigned probabilities greater than 0.5 associated with positive prediction. Model performance metrics were captured also using caret, and other described statistical tests were performed utilizing base R statistics package.

Codes utilized to conduct ML analysis can be found in GitHub, with the link below:

<https://github.com/msayer23/-Machine-Learning-Approaches-to-Predict-Early-Cardiac-Immune-Related-Adverse-Events->

**Sources:**

1. Data from: Trinetx Global Health Data Platform. 2023. Deposited 2013.

2. WHO Collaborating Centre for Drug Statistics Methodology. <https://atcddd.fhi.no/atc_ddd_index_and_guidelines/atc_ddd_index/>

3. Quan H, Li B, Couris CM, et al. Updating and validating the Charlson comorbidity index and score for risk adjustment in hospital discharge abstracts using data from 6 countries. *Am J Epidemiol*. Mar 15 2011;173(6):676-82. doi:10.1093/aje/kwq433

4. Quan H, Sundararajan V, Halfon P, et al. Coding algorithms for defining comorbidities in ICD-9-CM and ICD-10 administrative data. *Med Care*. Nov 2005;43(11):1130-9. doi:10.1097/01.mlr.0000182534.19832.83

5. team Rc. R: A language and environment for statistical computing. R Foundation for Statistical Computing. Vienna, Austria.

6. *Kuhn M*. The caret Package. <https://topepo.github.io/caret/index.html>

7. Lewis MJ, Spiliopoulou A, Goldmann K, Pitzalis C, McKeigue P, Barnes MR. nestedcv: an R package for fast implementation of nested cross-validation with embedded feature selection designed for transcriptomics and high-dimensional data. *Bioinform Adv*. 2023;3(1):vbad048. doi:10.1093/bioadv/vbad048

8. Kuhn M. Building Predictive Models in R Using the caret Package.

9. *fastshap: Fast Approximate Shapley Values.*

*R package*. Version version 0.1.1. 2024. <https://github.com/bgreenwell/fastshap>
